# Comparative Study on Alternative Splicing in Human Fungal Pathogens Suggests Its Involvement During Host Invasion

**DOI:** 10.3389/fmicb.2018.02313

**Published:** 2018-10-02

**Authors:** Patricia Sieber, Kerstin Voigt, Philipp Kämmer, Sascha Brunke, Stefan Schuster, Jörg Linde

**Affiliations:** ^1^Department of Bioinformatics, Faculty of Biological Sciences, Friedrich Schiller University, Jena, Germany; ^2^Research Group Systems Biology, Bioinformatics, Leibniz Institute for Natural Product Research and Infection Biology – Hans Knöll Institute, Jena, Germany; ^3^Jena Microbial Resource Collection, Leibniz Institute for Natural Product Research and Infection Biology – Hans Knöll Institute, Jena, Germany; ^4^Institute of Microbiology, Faculty of Biological Sciences, Friedrich Schiller University, Jena, Germany; ^5^Microbial Pathogenicity Mechanisms, Leibniz Institute for Natural Product Research and Infection Biology – Hans Knöll Institute, Jena, Germany; ^6^Research Group PiDOMICS, Leibniz Institute for Natural Product Research and Infection Biology – Hans Knöll Institute, Jena, Germany; ^7^Institute for Bacterial Infections and Zoonoses, Federal Research Institute for Animal Health–Friedrich-Loeffler-Institute, Jena, Germany

**Keywords:** alternative splicing, human fungal pathogens, RNA-Seq, comparative analysis, host-pathogen interaction

## Abstract

Alternative splicing (AS) is an important regulatory mechanism in eukaryotes but only little is known about its impact in fungi. Human fungal pathogens are of high clinical interest causing recurrent or life-threatening infections. AS can be well-investigated genome-wide and quantitatively with the powerful technology of RNA-Seq. Here, we systematically studied AS in human fungal pathogens based on RNA-Seq data. To do so, we investigated its effect in seven fungi during conditions simulating *ex vivo* infection processes and during *in vitro* stress. Genes undergoing AS are species-specific and act independently from differentially expressed genes pointing to an independent mechanism to change abundance and functionality. *Candida* species stand out with a low number of introns with higher and more varying lengths and more alternative splice sites. Moreover, we identified a functional difference between response to host and other stress conditions: During stress, AS affects more genes and is involved in diverse regulatory functions. In contrast, during response-to-host conditions, genes undergoing AS have membrane functionalities and might be involved in the interaction with the host. We assume that AS plays a crucial regulatory role in pathogenic fungi and is important in both response to host and stress conditions.

## 1. Introduction

**Figure 1:**
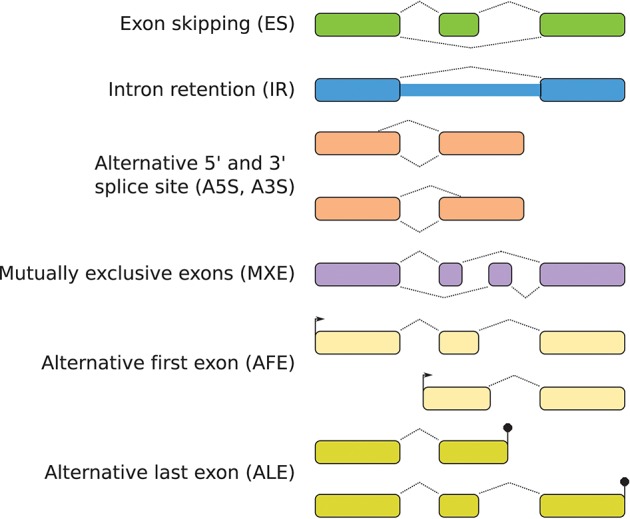
Patterns of alternative splicing (AS) result in different transcript isoforms.

Alternative splicing (AS) is an important regulatory mechanism of mRNA processing in eukaryotes resulting in multiple transcript isoforms of a single coding gene. It strongly increases the diversity and functional complexity of an organism and thus allows organisms to rapidly adapt to new environmental niches or stresses (Kelemen et al., 2013; Le et al., 2015). The spliceosome catalyzes the splicing process and excludes intronic sequences from pre-mature mRNA of genes with more than one exon (López et al., 2008; Le et al., 2015). This takes place by identifying, amongst others, the 5′ and 3′ splice sites of the exons. When the spliceosome detects alternative exon boundaries, AS takes place (Kelemen et al., 2013) with diverse possible AS patterns (Figure 1): Exon skipping (ES), intron retention (IR), alternative 5′ and 3′ splice site (A5S, A3S), alternative first exon or start site (AFE), alternative last exon or termination site (ALE), and mutually exclusive exons (MXE) (Le et al., 2015).

AS plays an important role in response to diverse environmental changes and is involved in the regulation of a wide range of cellular functions, including the inactivation of enzymes, changing binding affinity of proteins, and development of stem cells (Kelemen et al., 2013; Le et al., 2015; Schreiber et al., 2015). In human and mouse, AS mostly impacts the protein locally but not the general protein structure (Kelemen et al., 2013). A malfunction of AS can cause severe diseases such as cancer (Kelemen et al., 2013; Le et al., 2015).

Although the functionality of AS is well-studied in higher eukaryotes, its impact on fungi is only partially understood. It is known that AS occurs in various fungal species with a lower proportion of alternatively spliced genes compared to mammals (Kempken, 2013; Grützmann et al., 2014) but fungal pathogens may have a higher tendency to undergo AS than non-pathogenic fungi (Grützmann et al., 2014). Though only a small fraction of fungal species has been described to be pathogenic for humans (Advisory Committee on Dangerous Pathogens, 2013), those species are able to withstand the human immune system. Several of those species can cause severe systemic infections, which can be life-threatening especially for immunocompromised patients like transplant patients and those in intensive care units. Fungi of high clinical interest are for instance *Aspergillus fumigatus* with an estimated mortality rate up to 95%, and *Candida albicans*, which is predominant in hospital-acquired fungal bloodstream infections (Brown et al., 2012).

RNA-Seq data are most suitable for the analysis of AS by providing genome-wide information on exon coverage and splice junctions. It enables to distinguish and quantify the differential usage of splice patterns (Pan et al., 2008; Wang et al., 2008; Roberts et al., 2011; Trapnell et al., 2012b) originating from differentially expressed transcripts (DETs).

Several difficulties arise when analyzing AS in fungi. First, many genes and transcripts are not known or not yet annotated, and thus it is challenging to estimate their expression or identify their protein sequences and domains. Second, often only a single isoform is annotated, even though several exist. Third, the estimation of isoform expression is especially difficult when exons are part of multiple isoforms (Hooper, 2014) or when genes are overlapping. Further, the software tools for AS detection are mostly developed for higher organisms and do not perfectly fit the gene structure of fungi. Fourth, it is difficult to compare AS between different fungal species because there is only a small number of data sets available, and these are based on different experimental conditions.

**Figure 2:**
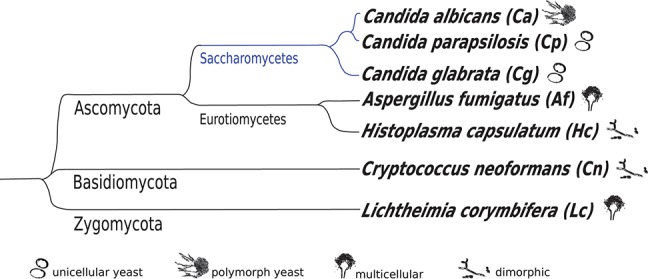
Phylogenetic tree of studied fungal species with corresponding morphology (Stajich et al., 2009). The branch of the studied *Candida* species is marked in blue to recognize the phylogeny in the following graphics.

In this study, we provide a comprehensive comparative transcriptomic analysis of AS in seven human-pathogenic fungi (Figure 2) based on RNA-Seq data. We investigate the impact of AS on response-to-host conditions and during environmental stresses by taking into account the evolutionary distance and morphology of the investigated fungal species. To this end, we compare AS patterns, splice sites and splicing efficiency on a genome-wide level. We detect DETs with four different AS software tools and compare the resulting genes with differentially expressed genes (DEGs). Further, we investigate the functional impact of AS in the studied species. *Candida* species show a special gene structure compared to the other studied species. In all fungi, IR is the most important AS pattern. Genes undergoing AS are species-specific and only 14% overlap with DEGs, which shows the importance to study AS additionally to differential gene expression. Here, we detect a number of AS events for genes involved in membrane function during response-to-host conditions, which suggest a general role of AS in the interaction with the host.

## 2. Materials and methods

### 2.1. Data sources

We used the classification of human fungal pathogens based on the list from (Advisory Committee on Dangerous Pathogens, 2013). Fungal species with a hazard group of 2 or higher were considered to be human-pathogenic. We based our analyses on RNA-Seq data from public available sources [Gene Expression Omnibus (GEO) (Edgar, 2002) and European Nucleotide Archive (Leinonen et al., 2011)] with data sets connected to response to the host (*ex vivo* interaction with human or murine cells, mostly immune cells) and other isolated stress conditions for the studied species. If data of more than one strain were available, we included those of the strain with the most experiments available. Mutant strains and experiments with less than two replicates were not considered. A complete list of utilized data sets is available in Table S1. In total, we compared seven fungal species (Figure 2). The corresponding GFF files and genome sequences are listed in Table S7.

### 2.2. Data preprocessing

We preprocessed all data with a standardized pipeline (Seelbinder et al., 2018) including a quality control using Fastqc v0.11.5 (Andrews, 2010), read trimming with Trimmomatic v0.32 (with a window size of 15 and a quality trimming threshold of 25) (Bolger et al., 2014), and read mapping with Tophat2 v2.1.0 (Kim et al., 2013) (with the parameters -g 1 –no-mixed –no-discordant –b2-very-sensitive, the GFF file for mapping guidance, and the parameters of minimal and maximal intron length adjusted).

### 2.3. Transcriptome prediction and abundance estimation

We predicted additional transcripts using Stringtie v1.3.3 (Pertea et al., 2015) following the protocol provided by (Pertea et al., 2016) and adapting the following parameters: -c 10 -m 30 -g 1. We merged the output using Stringtie (with the parameters -c 10 -F 1 -f 0.1 -g 1).

For each protein-coding gene with predicted isoforms, we checked whether the predicted isoform covered more than one gene or had no corresponding gene within the original annotation, and discarded it in these cases. We used the curated annotation to estimate the abundance of all genes and transcripts using Stringtie (Pertea et al., 2016). We applied an in-house script to determine possible AS patterns by comparing the remaining predicted isoforms after these processes of each gene to the originally annotated transcript isoform. This can be relevant if more than two isoforms are present as this assignment is relative to the reference isoform (Pohl et al., 2013). If there was more than one annotated isoform, we compared against the first annotated one, which is the most common one (Ezkurdia et al., 2015). Further, alternative exons and splice sites are known to have a lower affinity to the spliceosome and are less often recognized (Kelemen et al., 2013) and, thus, alternative transcripts have a lower expression level and usually are not or later annotated. The diploid annotation of *C. albicans* contains two copies of many genes, which were combined to compare the number of genes and isoforms to the other haploid species. For additional comparisons, we analyzed the genomic structure of the non-pathogenic yeast *S. cerevisiae* based on the annotation version R64-1-1.38 (Kersey et al., 2018).

### 2.4. Splice sites and splicing efficiency

We identified used splice sites using regtools v0.4.0 (https://regtools.readthedocs.io/en/latest/, and the genome-wide splicing efficiency for 5′ and 3′ splice sites applying the scripts provided by (Prevorovsky et al., 2016) based on predicted and annotated transcript isoforms. We checked the results exemplarily with IGV (Robinson et al., 2011) to ensure that the alternative splice sites are not single nucleotide variants but actual junction splice sites.

To compare the results to *S. cerevisiae*, we calculated the splicing efficiency for control and stress conditions for the GEO data sets GSE74361, GSE54825 and GSE85109 (Edgar, 2002).

### 2.5. Alternative splicing analysis

We based the AS analysis on four different tools to detect potential DETs: the R/Bioconductor package DEXSeq v1.22.1 (Anders et al., 2012), MATS v3.0.8 (Shen et al., 2014), MISO v0.5.2 (Katz et al., 2010) and DiffSplice v0.1.2beta (Hu et al., 2013), each with an FDR or *p*-value of 0.01 as threshold. Additional parameters for MISO are min-exon-size of 300 and Bayes factor threshold of 20, as suggested by (Katz et al., 2010). This analysis was performed separately for each data set.

We used the union of all potentially detected DETs and applied the following filter steps with the aim to remove false positive results: First, we removed genes without introns because AS takes place in genes with multiple exons (Le et al., 2015). Second, we excluded genes with a low expression of either an absolute coverage < 20 reads or a TPM value < 1 for each exon. Third, we allowed only junctions with the splice sites GT-AG, GC-AG, and AT-AC to identify actual 5′ and 3′ splice sites of internal exons (Brent, 2005). Further, we calculated the expression change pairwise between the exons, introns and junction counts of control and treatment. If the expression change was below a log2 fold change of 1, we considered it as a minor change and excluded it from further analyses.

We checked the remaining genes manually using IGV (Robinson et al., 2011) to identify genes with a change in expressed AS patterns between the compared conditions. To this end, we tested if there is a visible difference in used splice sites based on junction information, or a change in exon and intron expression. If the expression of an alternative pattern was below 10% compared to the originally annotated transcript isoform, we did not consider it. In addition, since we only used data with multiple replicates, we checked whether the observed AS event was visible in multiple replicates of a certain condition.

### 2.6. Differential gene expression analysis

We counted the mapped reads using FeatureCounts (Liao et al., 2014) based on the original GFF file without allowing multiple overlaps using the R/Bioconductor package Rsubread v1.18.0 (Liao et al., 2013). We applied the R/Bioconductor package DESeq2 v1.8.2 (Love et al., 2014) for DEG identification based on the counts from FeatureCounts and guided by the original GFF file comparing the control samples to the corresponding treatments as in the AS analysis (see Table S1). We considered genes with an adjusted *p* < 0.01 and an absolute fold change >2 as significantly differentially expressed. We classified significant DEGs with a TPM expression value < 1 per condition as not expressed and excluded them from our analyses. We compared the resulting DEGs to the predicted DETs of the corresponding data set.

Additionally, we identified genes involved in the spliceosomal activity from gene feature collections of Fungi Ensembl (Kersey et al., 2018) and *Candida* Genome Database (Skrzypek et al., 2017) (see Table S7, no annotated spliceosomal genes for the used strain of *H. capsulatum*). For this purpose, we searched for the term “splic” and excluded not relevant genes.

### 2.7. Orthologous and functional analyses

To identify orthologous genes between different *Candida* species, we extracted the classification of orthologous genes of all species from the *Candida* Genome Database (Skrzypek et al., 2017) and determined orthologs of predicted DETs between the studied *Candida* species. Further, we used the tool orthomcl v2.0.9 (Li, 2003) to predict orthologous groups of all considered species based on the corresponding protein sequences (listed in Table S7).

To gain information about underlying protein domains of specific genes, we used Interproscan v5.23-62.0 (Jones et al., 2014) based on the protein sequence of the considered transcript isoform. In addition, we applied TargetP v1.1 (Emanuelsson et al., 2007) to predict the location of the given protein sequence within the cell. We determined the GO terms of the predicted DETs using FungiFun2 (Priebe et al., 2015) based on the provided annotations from EBI and BLAST2GO. Since the number of genes was too low for an actual enrichment analysis and GO annotations of several genes are incomplete, we ranked the GO terms of DETs according to the number of occurrences separately for response-to-host and stress conditions in each species. We visualized the most common GO terms using Revigo (Supek et al., 2011) (see Figures S4–S12). Furthermore, we checked whether the resulting genes during response-to-host conditions are known in pathogen-host interaction, using the database PHI-base (Urban et al., 2015).

## 3. Results

### 3.1. Prediction of transcript isoforms for RNA-seq data of human fungal pathogens

Aiming at a comprehensive study of AS in human fungal pathogens, we obtained publicly available RNA-Seq experiments from several human fungal pathogens (Advisory Committee on Dangerous Pathogens, 2013) (see Methods for more details). We were able to include seven species in our analysis (Figure 2).

**Figure 3:**
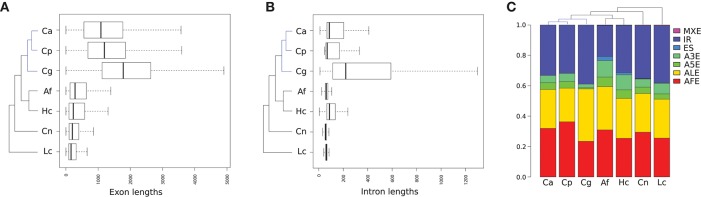
Boxplot of lengths distribution of exons **(A)** and introns **(B)** for all annotated and predicted transcripts of the studied fungal species are shown. The proportion of AS patterns for each fungal species **(C)** including mutually exclusive exons (MXE), intron retention (IR), exon skipping (ES), alternative 3′ splice site (A3S), alternative 5′ splice site (A5S), alternative last exon (ALE), alternative first exon (AFE). AS events of predicted isoforms are compared to the annotated isoforms. For abbreviations of fungal species, see Figure 2.

We mapped these data to the corresponding reference genome and predicted additional transcript isoforms to analyze AS including non-annotated splice variants. In all studied fungal species, we predicted new transcript isoforms (see Table 1). Ascomycota have fewer genes with multiple introns compared to *C. neoformans* and *L. corymbifera*. *Candida* species have the lowest number of genes and transcripts of the analyzed species and the individual genes contain fewer introns. Also, the relative number of intron-containing genes is much lower (below 10%) than for other studied fungal species (above 80%), as is the relative number of genes with multiple introns (below 1% vs. above 50% for other fungi) (Table 1). When comparing the lengths of introns and exons of the studied fungi (Figure 3), *Candida* species show a wider range including longer exons and introns. Introns of non-Ascomycota have comparable lengths without a high change (Figure 3B), unlike Ascomycota including yeasts with a high variety of intron lengths. We compared our results to the annotation of the model organism *S. cerevisiae* (Kersey et al., 2018). The number of intron-containing genes is low and similar to those in *Candida*. It has a similar genome size as other Saccharomycetes with 6692 genes and 358 introns, and no alternative isoforms are annotated. Also, the lengths of introns in *S. cerevisiae* vary comparable to the studied *Candida* species (see Figure S1).

**Table 1:** Statistical comparison of predicted and annotated isoforms for the investigated fungal species.

	**Species**	**Genes**	**Isoforms**	**Genes with multiple isoforms (%)**	**Introns**	**Genes with introns (%)**	**Genes with multiple introns (%)**	**Max intron per isoform**	**Median intron number per isoform**	**AS patterns per gene (relative)**
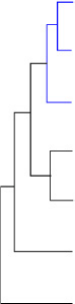	*C. albicans*	6,158	6,719	4.08	867	8.33	0.92	4	0	0.09
	*C. parapsilosis*	5,845	5,897	0.82	417	6.23	0.46	3	0	0.02
	*C. glabrata*	4,977	5,108	1.95	292	4.30	0.22	4	0	0.06
	*A. fumigatus*	10,144	13,879	30.21	30,312	80.74	54.45	25	2	0.96
	*H. capsulatum*	11,216	17,182	38.82	42,485	91.09	63.61	25	2	1.72
	*C. neoformans*	7,543	9,650	22.64	41,999	90.88	76.85	42	3	0.84
	*L. corymbifera*	11,350	14,015	18.98	54131	84.03	68.87	53	3	0.59

*The values of C. albicans refer to the haploid gene annotation*.

Further, we analyzed the occurence of the AS patterns (Figure 1) of originally annotated and predicted isoforms (Figure 3C). We observed a comparable proportion of AS patterns between all studied species. Intron retention (IR) is the most common pattern, followed by alternative first and last exons (AFE and ALE). The rate of alternative 3′ splice sites (A3S) is higher than that of alternative 5′ splice sites (A5S). Exon skipping (ES) and mutually exclusive exons (MXE) are almost missing.

### 3.2. Splice site usage and splicing efficiency under different conditions

We divided the available RNA-Seq data according to their treatments into the conditions groups “control”, “response-to-host conditions” and “stress” (see Table S1) to investigate the impact of these conditions on splicing-related characteristics.

**Figure 4:**
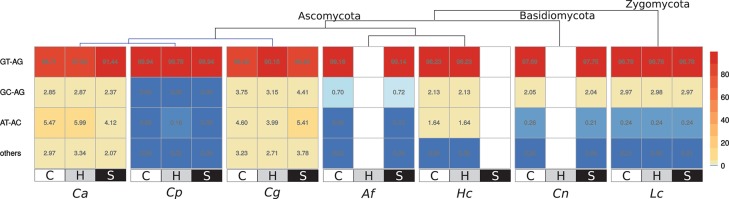
Used splice sites in the studied species (in %) during control (C), response-to-host (H) and stress (S) conditions for each species are shown. “Others” summarizes the ratio of the splice sites CT-AC, CT-GC, GN-AG, and GT-AT.

To identify the use of splice sites, we analyzed the 5′ and 3′ splice sites at the end of each intron (Figure 4). The canonical splice sites GT-AG appear most frequently with 88.32–99.89%, and the alternative splice sites GC-AG and AT-AC occur in 0.02–2.97 and 0.09–5.19%, respectively (average of all conditions of a fungal species). The frequency of the different splice sites does not change between control, stress and response-to-host conditions for each fungal species. However, in *C. albicans* and *C. glabrata*, the use of GT-AG is comparatively low with less than 90%; GC-AG and AT-AC are twice as often as in other fungi.

**Figure 5:**
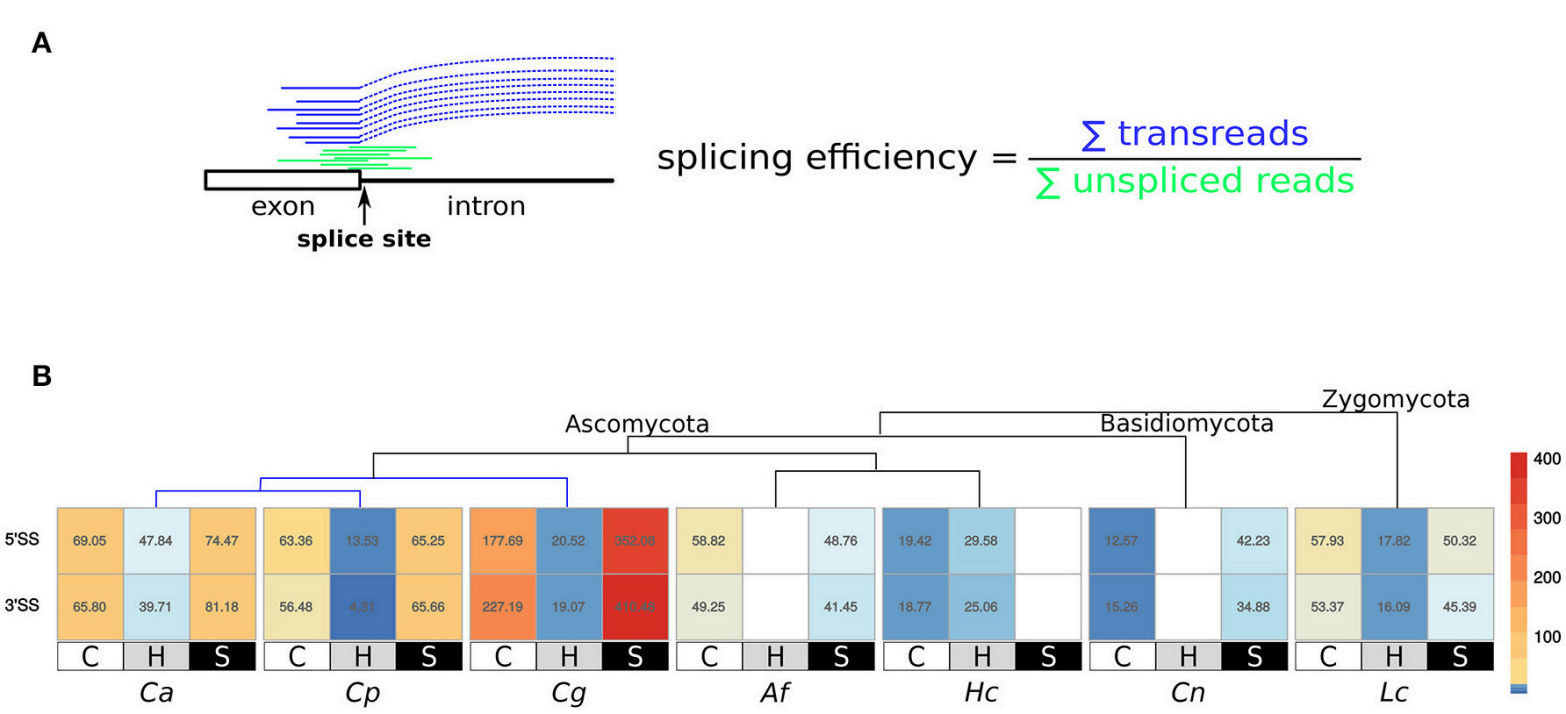
**(A)** The concept of splicing efficiency. For each splice site (SS), we calculated the number of transreads spanning the boundary between exon and intron (reads that are split at a specific splice site), and the number of reads covering the nearest intron position of the splice site (Prevorovsky et al., 2016). **(B)** Splicing efficiency during control (C), response-to-host (H) and other stress conditions (S) for each species. The values are summarized for all 5′ and 3′ SSs.

We identified the splicing efficiency to exclude intronic sequences under the different condition groups separately for 5′ and 3′ splice sites (Figure 5B). The splicing efficiency is very low in all studied species under response-to-host conditions compared to control and stress. In *C. glabrata* during control and stress conditions, the values are much higher than for the other studied fungi (more than 2- and 4-fold higher for control and stress, respectively).

### 3.3. Analyses of differentially alternatively spliced genes

In addition to the analysis of all expressed transcript isoforms, we identified differential AS, derived from differentially expressed transcripts (DETs), based on four different AS software tools (see Methods for more details). Again, we have distinguished between response-to-host and stress conditions and compared all data with the corresponding control.

**Figure 6:**
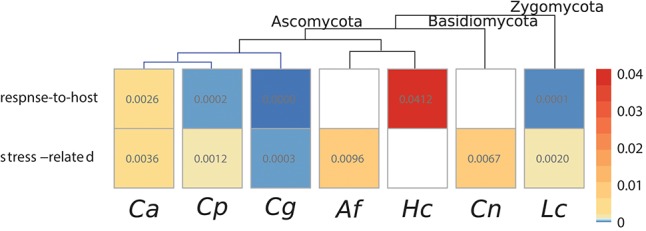
The number of predicted alternatively spliced genes relative to the number of genes. Response-to-host and stress conditions are compared to the corresponding control samples.

We excluded on average 22% of the genes due to a low coverage. Since genes in fungi are partially overlapping, AS detection is challenging and we inspected resulting genes manually to reject false positive results. In general, the expression of a certain isoform is not exclusive to a certain condition, but the rate between expressed isoforms changes. We found DETs for both stress and response-to-host conditions in a small number of genes (see Figure 6 and Table S3, S4). Only in *C. albicans*, there is an overlap between the sets of genes undergoing AS under response-to-host and other stress conditions (six common genes), in all the other cases they differ completely. Given the small size, we believe this overlap to be most likely circumstantial. Despite a few exceptions (4% of all DETs), detected genes appear only in one of the investigated data sets. The AS rate for stress is higher than for response-to-host conditions in all studied fungi when data sets are available for both conditions. We investigated the potential affect of AS in several genes by identifying the coded protein domains.

### 3.4. Interesting candidate genes undergoing alternative splicing

**Figure 7:**
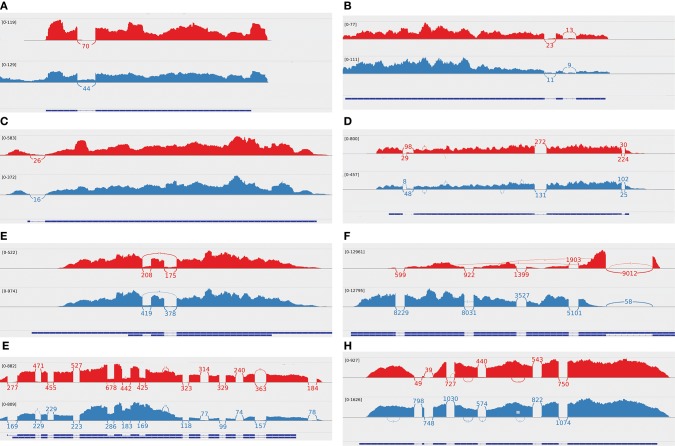
Sashimi plots of selected differentially alternatively spliced genes give information about gene expression (profile of mapped reads) and splice junctions (curved lines). Each plot shows the control (red) and the corresponding treatment (blue). The blue bars on the bottom indicate the exons, and the dotted lines indicate the introns of the corresponding gene. **(A)** *C. albicans SOD3* (manganese-containing superoxide dismutase) is shown during control (red) and oxidative stress (blue), which retains an intron. It appears similar during hyphal growth. **(B)** *C. albicans CAN3* (transmembrane amino acid transporter) is shown during control (red) and treatment with acetic acid (blue). It retains one or two introns during the stress treatment. **(C)** *C. parapsilosis CPAR2_801460* (glutathione transferase activity) is shown during control (red) and in a glucose-rich BMW medium (blue). It retains an intron during the control treatment. **(D)** *A. fumigatus AFUB_001340* (putative nuclear splicing factor) is shown during control (red) and treatment with caspofungin (blue) with alternative 3′ or 5′ splice sites. **(E)** *A. fumigatus AFUB_043270* (putative C6 transcription factor) is shown during control (red) and reduced oxygen for 15 minutes (blue). The portion of intron retention is higher during the control condition. **(F)** *C. neoformans CNAG_06101* (ADP/ATP carrier protein) is shown during control (red) and increased temperature (blue). It retains an intron during the treatment within the 5′ UTR. **(G)** *L. corymbifera LCOR_03517.1* (transmembrane protein) is shown during control (red) and hypoxia (blue) with three retained introns. **(H)** *L. corymbifera LCOR_10856.1* (sugar transporter) is shown during control (red) and iron depletion (blue) with an alternative start site after the first two exons.

When investigating genes undergoing AS, we identified several examples that may have a crucial functional impact: During infection, pathogenic fungi are faced with oxidative stress originating from host cells. To combat these toxic molecules, fungi express several genes coding for detoxifying enzymes like catalases or superoxide dismutases. *C. albicans* is known to up-regulate particular superoxide dismutases depending on the circumstances of the environment, e. g. *SOD5* is highly up-regulated during human blood infection (Fradin et al., 2005). Superoxide dismutases contain different trace metals (SOD1: copper and zinc, SOD3: manganese) enabling the degradation of superoxide to hydrogen peroxide or molecular oxygen (Mayer et al., 2013; Cuéllar-Cruz et al., 2014; Urban et al., 2015). We found *C. albcians* superoxide dismutases *SOD1* and *SOD3* being alternatively spliced during stress and response-to-host conditions (during oxidative stress, treatment with weak organic acids and hyphal growths). The alternative isoform of *SOD3* has a retained intron and is differentially expressed under both stress and response-to-host conditions (Figure 7A), and misses its C-terminal domain and manganese binding site. The gene *SOD1* has an alternative start site during treatment with different weak organic acids resulting in a non-functional amino acid sequence caused by a premature stop codon. This indicates that AS may be another mechanism to regulate superoxide dismutase activity due to changing availability of trace metals in different environmental conditions. Also, under weak organic acetic acid stress, induced by acetic acid, the transmembrane amino acid transporter gene *CAN3* retains one or both introns (Figure 7B). If only the first exon is kept, a premature stop codon is introduced. However, if this gene retains both introns, a different start codon located within the second intron becomes available. This potential protein misses a conserved site of the amino acid permease but contains all functional domains, which may be a mechanism to adapt to the lack of nutritive substance under acid stress.

In *C. parapsilosis*, genes known to be up-regulated during interaction with immune cells are alternatively spliced (Wilson et al., 2009). The gene *CPAR2_801460*, which codes for a protein with glutathione transferase activity, is involved in the response to reactive oxygen species in *C. albicans* and retains its intron during stress (Figure 7C). The corresponding amino acid sequence has two protein domains less compared to the main transcript but is not completely non-functional.

The *A. fumigatus* gene *AFUB_001340* (Figure 7D) encoding a putative nuclear splicing factor undergoes AS during treatment with the antifungal drug caspofungin, which attacks the fungal cell wall (Brown et al., 2013). The two additional isoforms with either a modified 3′ SS or 5′ SS lose only a single domain, but not the full function. These alternative transcripts might still be functioning normally or might have an altered function to react to the cell wall effects of caspofungin. This shows that AS can affect also the splicing mechanism itself although only a small number of genes involved in splicing processes is alternatively spliced. Another example is the transcription factor *AFUB_043270* (Figure 7E), which has less retained introns in a low oxygen environment. The protein encoded by the original transcript contains Zn(2)-C6 DNA-binding domains, which are not encoded in the alternative transcript. AS may be a mechanism to control the activation of transcription factor *AFUB_043270* during low oxygen supply, which is less active under control treatment.

The gene *CNAG_06101* of *C. neoformans* codes for a mitochondrial ADP/ATP carrier protein. Under high temperature stress, an intron within the 5′ UTR is retained (Figure 7F). Based on the predictions of TargetP (Emanuelsson et al., 2007), the original annotated transcript is a mitochondrial targeting peptide, which is not the case for the alternative transcript and can have a regulatory effect.

The gene *LCOR_03517.1* in *L. corymbifera* codes for a protein with a transmembrane activity and retains three introns in combination under hypoxic conditions (Figure 7G). This alternative isoform has the same protein domains and remains functional. This is a rare case, as IR mostly causes a frame shift that is likely to cause degradation (Grützmann et al., 2014). In addition, it is known that retained introns may contain regulatory elements (Kelemen et al., 2013; Kawashima et al., 2014). The alternative transcript may possess an additional regulatory importance without loss of the original function. We assume that AS is important in fungi for adaption and stress tolerance via the generation of suitable splice variants. A further interesting candidate gene of this species is the sugar transporter gene *LCOR_10856.1* (Figure 7H) with an alternative start site. Interestingly, the shorter alternative transcript codes for the same protein domains but lacks a putative signal preptide of the full transcript (lower reliability class predicted using TargetP).

### 3.5. Relation with differentially expressed and orthologous genes

The detected DETs have only a low overlap with DEGs and genes involved in the spliceosomal regulation. Spliceosomal genes overlap with only 0.8% of all DETs and only during stress conditions and differ between the data sets. DEGs and DETs overlap for 14% of the genes (Table S6).

Based on known orthologous genes between the studied *Candida* species, we investigated common genes undergoing AS resulting in no overlap of orthologous genes in the set of DETs between these closely related species. A further prediction of orthologous gene groups based on the protein sequences of all studied species confirms these results: Not a single identified DET has orthologs in the set of DETs of all investigated species.

We determined the corresponding GO terms of the predicted DETs to measure the functional impact of AS (see Figures S4–S9). The identified terms during stress conditions vary widely and the respective genes are involved in various cellular functions. During response-to-host conditions, all comparisons with available GO terms contain “membrane” or “membrane regulation.” In *C. albicans*, we identified the GO terms “pathogenesis” and “filamentous growth” during response-to-host conditions. Results of *L. corymbifera* contain the term “fungal-type cell wall.”

## 4. Discussion

### 4.1. Comprehensive investigation for RNA-seq data of human fungal pathogens

The majority of pathogenic fungi with available RNA-Seq data are Ascomycota including three closely related *Candida* species (Figure 2). The investigated fungal species are those with a broad research community and are the most important candidates of severe fungal infections (e.g. lung infections caused by *A. fumigatus* and bloodstream infections caused by *Candida* species) (Brown et al., 2012).

For most fungi, the annotation of transcript isoforms is not complete and often only one transcript isoform per gene is annotated (Schreiber et al., 2015). To determine the potential capacity of AS, it was necessary to predict alternative isoforms (see Methods for more details). Furthermore, publicly available data are not directly comparable to each other due to different measured time points and treatments. To overcome this problem, we grouped the samples according to the more broadly defined condition groups “control” (mock treatments), “response-to-host conditions” (interaction with *ex vivo* human or murine cells, mostly immune cells to simulate infection) and “stress” (other *in vitro* stress conditions and isolated stress response such as change in temperature, pH value, and oxygen levels).

### 4.2. Prediction of transcript isoforms reveals a particular role of *Candida* species

We used the predicted and originally annotated transcript isoforms to investigate a genome-wide survey of splicing. For this purpose, we tested three mapping software tools and four genome-guided transcriptome assembly tools that are well applicable for small genomes (Steijger et al., 2013; da Fonseca et al., 2016). Regarding mapping software, we tested the tools in combination with AS analysis tools and decided to use Tophat2 because it results in more potential genes undergoing AS compared to other tested tools (data not shown). Although this may include false positive results, it reduces the chance to exclude potential genes undergoing AS. Further, we decided to use the transcriptome assembly tool Stringtie as it shows good performance for genes with low abundance and multiple isoforms in comparative studies and is suitable for non-model organisms (Pertea et al., 2015; da Fonseca et al., 2016). Additionally, Stringtie predicts IR best compared to other tested tools, which is beneficial for AS prediction in fungi. Other assembly tools did not meet our requirements: Since we combined all available RNA-Seq data for a species, the computing workload is high and caused errors using iReckon (Mezlini et al., 2012). Cufflinks2 (Trapnell et al., 2012a) computed a high number of transcripts per gene, which is most likely an overestimate for fungal transcriptomes. The multiple read lengths of different experiments were not suitably using SLIDE (Li et al., 2011).

We predicted new transcript isoforms for all observed fungi (Table 1) and observed that Ascomycota have fewer genes with multiple introns compared to other investigated fungi. *Candida* species are characterized by the lowest number of genes and transcripts and the individual genes contain fewer introns. In addition, *Candida* species show higher variability of intron and exon lengths of the studied fungi including longer exons and introns (Figure 3). The partially longer introns of *Candida* genes might contain more regulatory elements such as small nucleolar RNAs, which are known to be frequently located within introns in yeasts (Donovan et al., 2016). In general, *Candida* species have a smaller genome and intron number but a more varying intron and exon structure and stand out in this comparison. We compared our results to the model organism *S. cerevisiae*, which is closely related to *Candida* species. The number and lengths of exons and introns are comparable to those in the studied *Candida* species. This shows that our results are not specific for *Candida* species but likely rather valid for Saccharomycetes in general. Furthermore, the results for the different *Candida* species and *S. cerevisiae* are similar despite the differences in conditions and number of available data sets. This indicates that our findings are not strongly affected by these differences in the publicly available data. The number of predicted AS events relative to the gene number vary essentially between all studied fungi (see the last column of Table 1). This value is very low for *Candida* species due to the low number of genes containing introns. *H. capsulatum* is the only studied species with on average more than one AS pattern per gene, which is associated with the highest proportion of genes with multiple isoforms. For all fungi, we observe a correlation between the predicted numbers of AS events and isoforms per gene. We conclude that regularly one AS event and not a combination of multiple events is responsible for each alternative isoform.

### 4.3. Intron retention and alternative first and last exons are the most common splicing patterns in human fungal pathogens

In addition to the number of isoforms, the type of splicing events can differ between species. We investigated these differences by comparing the AS patterns between originally annotated and predicted isoforms (see Figures 1, 3C). The proportion of AS patterns is similar between all studied species: Intron retention (IR) is the most occurring pattern followed by alternative first and last exons (AFE and ALE). With these results, our data add further evidence to the finding that IR is generally the most prevalent splicing pattern in fungi (Kempken, 2013; Grützmann et al., 2014; Schreiber et al., 2015). The identification of AFE and ALE as important AS patterns confirms previous studies in *S. cerevisiae* (Schreiber et al., 2015). The high rate of alternative gene boundaries may arise from unannotated UTR regions, which are covered by reads and thus are recognized by Stringtie. Further, a higher rate of alternative 3′ splice sites (A3S) than 5′ splice sites (A5S) has been detected before for fungal species based on expressed sequence tags (ESTs) (Kempken, 2013). Here, we confirm this behavior based on the genome-wide approach of RNA-Seq data. Exon skipping (ES) occurs at a rate of less than 1% in all fungi and mutually exclusive exons (MXE) are almost absent (below 0.2%), which is in agreement with previous studies (Kempken, 2013). MXE was found to play a role in higher eukaryotes (Pohl et al., 2013) but might not be relevant in fungi. In vertebrates including humans, IR plays a minor role with only 3% and ES is the major AS pattern (Keren et al., 2010; Le et al., 2015). In contrast, the proportion of IR and ES in fungi is comparable to those in plants, although fungi are more closely related to animals (Mcguire et al., 2008). This shows that AS patterns are not conserved between different but within phylogenetic kingdoms. As our results are similar for the different fungal species and confirm previous findings, the comparability of the different used data sets and treatments between the studied species does not have an extensive effect.

### 4.4. More alternative splice sites in *C. albicans* and *C. glabrata*

As the AS patterns differ to those in vertebrates, we next studied the use of splice sites. To this end, we analyzed the 5′ and 3′ splice sites at the boundaries between exons and introns (Figure 4). The most common are the canonical splice sites GT-AG, the alternative splice sites GC-AG and AT-AC occur much less frequently. In human genes, they appear in a comparable proportion as in fungi with GT-AG as most prevalent with 98.93%, followed by GC-AG with 0.89% and AT-AC with 0.10% (Parada et al., 2014).

The used splice sites do not differ between control, stress and response-to-host conditions for any fungal species. However, in *C. albicans* and *C. glabrata*, the usage of GT-AG is unusual low and the alternative splice sites occur twice as frequently as in other fungal species. Kawashima and colleagues have identified this behavior of alternative splice sites in *S. cerevisiae*, which often results in non-functional transcripts and may indicate a regulatory role to control the overall biosynthesis of the functional protein of the corresponding gene (Kawashima et al., 2014). Alternative splice sites are recognized by the minor spliceosome, which contains the subunits U11, U12, U4atac, and U6atac (López et al., 2008). However, these components of the minor spliceosome have not been identified in any of the studied fungi. Some components have been predicted in close relatives of *L. corymbifera* and might be present in some members of the Zygomycota. In contrast, there is no evidence of the minor spliceosome in yeast species (López et al., 2008). We speculate that a minor spliceosome in yeasts exists but may be compromised of different proteins than in other eukaryotes.

### 4.5. Lower splicing efficiency during response-to-host conditions

In the next step, we determined the functionality of the splicing machinery by calculating the splicing efficiency (Figure 5A) (Prevorovsky et al., 2016). Splicing efficiency is the proportion of spliced transreads (number of junctions spanning the splice site) to unspliced reads (reads covering the first/last position of the intron at the splice site) calculated for each 5′ and 3′ splice site in the genome (Figure 5B).

During response-to-host conditions, the splicing efficiency is very low in all studied species, which means that there are more unspliced reads. In *C. glabrata*, the splicing efficiency during growth under control and stress conditions is much higher than in all other fungi. We compared these results to the splicing efficiency in *S. cerevisiae*. This non-pathogenic species behaves unlike *C. glabrata* and shows comparable values to the other studied species.

The higher rate of unspliced reads at a certain splice site during response-to-host conditions can be caused by IR, A3S and A5S. We assume that IR is the main cause of this observation because it is one of the predominant AS patterns and the values for 5′ and 3′ splice sites are balanced. The low splicing efficiency could indicate that the splicing machinery does not work properly during interaction with host cells, resulting in generalized splicing disorders, or/and that a specifically higher rate of IR takes place as a response to host conditions. To investigate possible reasons for the change in splicing efficiency, we determined the expression of genes, including those involved in spliceosomal regulation. Surprisingly, there is neither a difference in the number of all expressed genes or transcripts between the compared condition groups (see Table S2 and Figure S2), nor a difference in the number of detected differentially expressed genes (DEGs) (see Table S6). Almost all spliceosomal genes are expressed in all studied data sets of all conditions. The spliceosomal genes that are differentially expressed are all up-regulated during both response-to-host and other stress conditions, which shows that the spliceosomal activity is increased compared to the control condition. We assume that the lower splicing efficiency likely does not originate from the repressed spliceosome but may be caused by a different activity of associated splicing factors. However, we cannot ensure whether the spliceosome is working correctly and whether IR is an induced process.

### 4.6. AS acts independently from DEGs and at higher rates under stress conditions

In contrast to the analysis of all isoforms, we detected alternatively spliced genes resulting in differentially expressed transcripts (DETs) for response-to-host and stress conditions compared to the corresponding control based on four different tools. Since the AS tools rely on different levels of prediction (exon level, gene level, exon and junction level) (Hooper, 2014), the results differ strongly.

For both stress and response-to-host conditions, we detected DETs for a low number of genes (see Figure 6). In all studied fungi with data sets of both conditions, the AS rate is higher for stress than for response-to-host conditions, although the number of experiments varies between the species. We are aware that the different numbers of data sets and treatments for the studied species may have an impact and that additional data might result in more DETs. This could be an explanation for the higher proportion of AS in *C. albicans* compared to *C. glabrata* and *C. parapsilosis*. However, the number of DETs is higher during stress conditions for all fungi and shows a common tendency independent of the number of considered treatments. We assume that AS plays a higher regulatory role during stress in all fungal species, which supports similar findings in *S. cerevisiae* (Kawashima et al., 2014; Schreiber et al., 2015). Although our analysis seemed robust against the differences in treatment inherent to using publicly available data, an analysis of data sets of different fungi exposed to the same treatment could increase its accuracy and explanatory power. Such data are available for *Candida* species in a co-culture with blood (GSE114174-7) and have been included in this analysis, but no similar set is available for fungi of other phyla yet.

Non-yeast fungal species have more intron-containing genes and, thus, a higher potential to undergo AS. However, their number of DETs is not higher than in *Candida* species, which shows that AS is an important regulatory mechanism also in species with a low number of intron-containing genes. The ratio of AS patterns per gene is above one for *H. capsulatum* (292 AS patterns in 236 genes), which agrees with the results predicted by Stringtie with a higher number of transcript isoforms per gene in this species (see the last column of Table 1, Tables S4, S5). Furthermore, the detected AS patterns and the results predicted by Stringtie agree with each other with IR as being the predominant AS pattern in all fungi, and AFE and A3S with a high frequency (see Table S5 and Figure S3). Due to the low number of events, the possible difference between AS patterns during response-to-host and stress conditions is not sufficient for a reliable conclusion.

We went on to study the common regulation of detected DETs with DEGs and genes involved in the spliceosomal regulation (see Table S6): Spliceosomal genes have less than 1% overlap with the detected DETs, indicating that the regulation of AS and spliceosome have only a minor correlation. Differential expression at DET and DEG level overlap for 14% of the genes. Thus, in most instances, AS acts independently from DEGs and AS analyses can give important additional information about regulatory mechanisms.

### 4.7. AS is more likely to have a regulatory rather than a protein-coding function

We observed several candidate genes with potential important regulatory effects during response-to-host or stress conditions, as shown in detail in section 3.4 and Figure 7. None of the presented candidate genes has been identified as DEG and might be overlooked in experiments where AS is not investigated. The functional impact is not completely clear. It is known that AS in fungi can lead to non-functional isoforms, which can be a mechanism to regulate the overall expression of a gene (Kempken, 2013; Grützmann et al., 2014; Kawashima et al., 2014; Schreiber et al., 2015). Also, we might miss functional information of fungal genes due to undiscovered additional protein domains that influence the protein function. Furthermore, several selected candidate genes remain functional and have the potential for additional regulatory functionality, which might be an adaption to the environmental changes, confirming the need to analyze the effect of AS in fungal species. It should be noted that RNA-Seq data represent the transcript level, which can differ from the protein level. This means that alternative transcripts may not be translated into the corresponding proteins, in case the transcript encodes for transcript is protein-coding. This applies in particular to dying cells, where transcript levels decrease in general, and, thus, AS might not be measurable on the protein level.

### 4.8. AS is species-specific, but has a common functional tendency during response-to-host conditions

Based on orthologous genes that are known between *Candida* species and predicted for all species studied, we found that none of the detected DETs has orthologues in DETs of the other studied fungi. This shows that the influence of AS on certain genes is not conserved between the species. A species-specific observation is needed to gain more insights into the influence of AS in an individual species.

To estimate the functional impact of AS, we determined the underlying GO terms of the predicted DETs (see Figures S4–S12). During stress, the identified terms differ strongly and the underlying genes are involved in diverse cellular functions. This leads us to the conclusion that AS may be involved in various cellular mechanisms during the response to stress (Schreiber et al., 2015). In contrast, it is noteworthy that under all response-to-host conditions with available GO terms “membrane” or “membrane regulation” appear, which might be due to host recognition and adhesion processes (Moran et al., 2010). Additionally in *C. albicans*, the GO terms “pathogenesis” and “filamentous growth” were discovered during response-to-host conditions in DETs and support previous studies (Wilson et al., 2009). Furthermore in *L. corymbifera*, the term “fungal-type cell wall” was detected, which allows the assumption that this species attempts to adapt intracellularly to the response-to-host condition by an increased expression of splice variants of genes involved in the fungal cell wall. In summary, these results suggest that AS may be directly involved in host invasion and support the theory that AS plays a role during response-to-host conditions in pathogenic fungi (Grützmann et al., 2014).

The investigated stress conditions occur as part of infection processes, e. g. oxidative or nitrosative stress also play a role during infection (Brown et al., 2013; Cuéllar-Cruz et al., 2014). However, we see a clear difference between the response to *ex vivo* stress and complex infection processes. This is possibly related to the response-to-host treatment, which is a combination of different stresses (Brown et al., 2013).

## 5. Conclusions

Alternative splicing (AS) and its impact are rarely studied in human fungal pathogens during response-to-host processes. Comparative genome-wide surveys of AS in fungi have been carried out before using expressed sequence tags (ESTs) (Grützmann et al., 2014). Here, we performed a systematic evaluation of AS in seven pathogenic fungi based on well established RNA-Seq data and compared its effect on response-to-host and other stress conditions. We predicted alternatively isoforms and detected differentially expressed transcripts in a genome-wide manner.

We found that *Candida* yeast species differ in the number and size of introns compared to other studied species. In all fungi, intron retention (IR) and alternative first and last exons (AFE, ALE) are the major AS patterns. Mostly, AS acts independently of differential gene expression, which shows that AS analyses provide additional information to DEGs. We identified genes undergoing AS with a functional alternative transcript and/or a potential regulatory impact. AS affects genes species-specifically and DETs are not conserved between the different studied fungi. However, there is a common functional influence of affected genes: AS occurs more frequently under stress conditions and affects genes of various regulatory functions. Under response-to-host conditions, AS rate and splicing efficiency are lower and AS plays an important role in regulating the cell membrane. This suggests that the functional impact of AS during response-to-host conditions may be involved in the interaction with host cells and is important for adaptation during the interaction with the host.

## Data availability statement

The data analyzed for this study can be found in the GEO and are listed in Table S1.

## Author contributions

PS conducted all analyses and visualizations. The paper structure and content was established in cooperation with JL. PK, SB, and KV provided additional data to this comparative study. Interpretation of first results and further steps were discussed with JL, KV, and SS. Biological interpretation was conducted and interesting candidate genes for specific investigation were extracted by PK, SB, and KV. All authors contributed in writing the manuscript.

### Conflict of interest statement

The authors declare that the research was conducted in the absence of any commercial or financial relationships that could be construed as a potential conflict of interest.

## References

[B1] Advisory Committee on Dangerous Pathogens (2013). The Approved List of Biological Agents, 3rd Edn. (London: Health and Safety Executive) Available online at: http://www.hse.gov.uk/pubns/misc208.htm

[B2] Anders S. Reyes A. Huber W. (2012). Detecting differential usage of exons from RNA-seq data. Genome Res. 22, 2008–2017. 10.1101/gr.133744.111 22722343 PMC3460195

[B3] Andrews S. (2010). Fastqc: A Quality Control Tool for High Throughput Sequence Data. Babraham Bioinformatics-Fastqc A Quality Control Tool for High Throughput Sequence Data.

[B4] Bolger A. M. Lohse M. Usadel B. (2014). Trimmomatic: a flexible trimmer for Illumina sequence data. Bioinformatics 30, 2114–2120. 10.1093/bioinformatics/btu170 24695404 PMC4103590

[B5] Brent M. R. (2005). Genome annotation past, present, and future: how to define an ORF at each locus. Genome Res. 15, 1777–1786. 10.1101/gr.3866105 16339376

[B6] Brown A. J. Budge S. Kaloriti D. Tillmann A. Jacobsen M. D. Yin Z.. (2013). Stress adaptation in a pathogenic fungus. J. Exp. Biol. 217, 144–155. 10.1242/jeb.088930 24353214 PMC3867497

[B7] Brown G. D. Denning D. W. Gow N. A. Levitz S. M. Netea M. G. White T. C. (2012). Hidden killers: human fungal infections. Sci. Trans. Med. 4, 165rv13–165rv13. 10.1126/science.1222236 23253612

[B8] Cuéllar-Cruz M. López-Romero E. Ruiz-Baca E. Zazueta-Sandoval R. (2014). Differential response of *Candida albicans* and *Candida glabrata* to oxidative and nitrosative stresses. Curr. Microbiol. 69, 733–739. 10.1007/s00284-014-0651-3 25002360

[B9] da Fonseca R. R. Albrechtsen A. Themudo G. E. Ramos-Madrigal J. Sibbesen J. A. Maretty L.. (2016). Next-generation biology: sequencing and data analysis approaches for non-model organisms. Mar. Genomics 30, 3–13. 10.1016/j.margen.2016.04.012 27184710

[B10] Donovan P. D. Schröder M. S. Higgins D. G. Butler G. (2016). Identification of non-coding RNAs in the *Candida parapsilosis* species group. PLoS oNE 11:e0163235. 10.1371/journal.pone.0163235 27658249 PMC5033589

[B11] Edgar R. (2002). Gene Expression Omnibus: NCBI gene expression and hybridization array data repository. Nucleic Acids Res. 30, 207–210. 10.1093/nar/30.1.207 11752295 PMC99122

[B12] Emanuelsson O. Brunak S. von Heijne G. Nielsen H. (2007). Locating proteins in the cell using TargetP, SignalP and related tools. Nat. Protocols 2, 953–971. 10.1038/nprot.2007.131 17446895

[B13] Ezkurdia I. Rodriguez J. M. Carrillo-de Santa Pau E. Vázquez J. Valencia A. Tress M. L. (2015). Most highly expressed protein-coding genes have a single dominant isoform. J. Proteome Res. 14, 1880–1887. 10.1021/pr501286b 25732134 PMC4768900

[B14] Fradin C. De Groot P. MacCallum D. Schaller M. Klis F. Odds F. C. (2005). Granulocytes govern the transcriptional response, morphology and proliferation of *Candida albicans* in human blood. Mol. Microbiol. 56, 397–415. 10.1111/j.1365-2958.2005.04557.x 15813733

[B15] Grützmann K. Szafranski K. Pohl M. Voigt K. Petzold A. Schuster S. (2014). Fungal alternative splicing is associated with multicellular complexity and virulence: a genome-wide multi-species study. DNA Res. 21, 27–39. 10.1093/dnares/dst038 24122896 PMC3925392

[B16] Hooper J. E. (2014). A survey of software for genome-wide discovery of differential splicing in RNA-Seq data. Hum. Genomics 8:3. 10.1186/1479-7364-8-3 24447644 PMC3903050

[B17] Hu Y. Huang Y. Du Y. Orellana C. F. Singh D. Johnson A. R.. (2013). DiffSplice: the genome-wide detection of differential splicing events with RNA-seq. Nucleic Acids Res. 41, e39. 10.1093/nar/gks1026 23155066 PMC3553996

[B18] Jones P. Binns D. Chang H. Y. Fraser M. Li W. McAnulla C.. (2014). Interproscan 5: genome-scale protein function classification. Bioinformatics 30, 1236–1240. 10.1093/bioinformatics/btu031 24451626 PMC3998142

[B19] Katz Y. Wang E. T. Airoldi E. M. Burge C. B. (2010). Analysis and design of RNA sequencing experiments for identifying isoform regulation. Nat. Methods 7, 1009–1015. 10.1038/nmeth.1528 21057496 PMC3037023

[B20] Kawashima T. Douglass S. Gabunilas J. Pellegrini M. Chanfreau G. F. (2014). Widespread use of non-productive alternative splice sites in *Saccharomyces cerevisiae*. PLoS Genet. 10:e1004249. 10.1371/journal.pgen.1004249 24722551 PMC3983031

[B21] Kelemen O. Convertini P. Zhang Z. Wen Y. Shen M. Falaleeva M. Stamm S. (2013). Function of alternative splicing. Gene 514, 1–30. 10.1016/j.gene.2012.07.083 22909801 PMC5632952

[B22] Kempken F. (2013). Alternative splicing in ascomycetes. Appl. Microbiol. Biotechnol. 97, 4235–4241. 10.1007/s00253-013-4841-x 23515838

[B23] Keren H. Lev-Maor G. Ast G. (2010). Alternative splicing and evolution: diversification, exon definition and function. Nat. Rev. Genet. 11, 345–355. 10.1038/nrg2776 20376054

[B24] Kersey P. J. Allen J. E. Allot A. Barba M. Boddu S. Bolt B. J.. (2018). Ensembl genomes 2018: an integrated omics infrastructure for non-vertebrate species. Nucleic Acids Res. 46, D802–D808. 10.1093/nar/gkx1011 29092050 PMC5753204

[B25] Kim D. Pertea G. Trapnell C. Pimentel H. Kelley R. Salzberg S. L. (2013). Tophat2: accurate alignment of transcriptomes in the presence of insertions, deletions and gene fusions. Genome Biol. 14:R36. 10.1186/gb-2013-14-4-r36 23618408 PMC4053844

[B26] Le K. Q. Prabhakar B. S. Hong W. J. Li L. C. (2015). Alternative splicing as a biomarker and potential target for drug discovery. Acta Pharmacol. Sin. 36, 1212–1218. 10.1038/aps.2015.43 26073330 PMC4648177

[B27] Leinonen R. Akhtar R. Birney E. Bower L. Cerdeno-Tárraga A. Cheng Y.. (2011). The european nucleotide archive. Nucleic Acids Res. 39(suppl. 1), D28–D31. 10.1093/nar/gkq967 20972220 PMC3013801

[B28] Li J. J. Jiang C. R. Brown J. B. Huang H. Bickel P. J. (2011). Sparse linear modeling of next-generation mRNA sequencing (RNA-Seq) data for isoform discovery and abundance estimation. Proc. Natl. Acad. Sci. U.S.A. 108, 19867–19872. 10.1073/pnas.1113972108 22135461 PMC3250192

[B29] Li L. (2003). Orthomcl: identification of ortholog groups for eukaryotic genomes. Genome Res. 13, 2178–2189. 10.1101/gr.1224503 12952885 PMC403725

[B30] Liao Y. Smyth G. K. Shi W. (2013). The Subread aligner: fast, accurate and scalable read mapping by seed-and-vote. Nucleic Acids Res. 41:e108. 10.1093/nar/gkt214 23558742 PMC3664803

[B31] Liao Y. Smyth G. K. Shi W. (2014). featurecounts: an efficient general purpose program for assigning sequence reads to genomic features. Bioinformatics 30, 923–930. 10.1093/bioinformatics/btt656 24227677

[B32] López M. D. Rosenblad M. A. Samuelsson T. (2008). Computational screen for spliceosomal RNA genes aids in defining the phylogenetic distribution of major and minor spliceosomal components. Nucleic Acids Res. 36, 3001–3010. 10.1093/nar/gkn142 18390578 PMC2396436

[B33] Love M. I. Huber W. Anders S. (2014). Moderated estimation of fold change and dispersion for RNA-seq data with DESeq2. Genome Biol. 15:550. 10.1186/s13059-014-0550-8 25516281 PMC4302049

[B34] Mayer F. L. Wilson D. Hube B. (2013). *Candida albicans* pathogenicity mechanisms. Virulence 4, 119–128. 10.4161/viru.22913 23302789 PMC3654610

[B35] Mcguire A. M. Pearson M. D. Neafsey D. E. Galagan J. E. (2008). Cross-kingdom patterns of alternative splicing and splice recognition. Genome Biol. 9:R50. 10.1186/gb-2008-9-3-r50 18321378 PMC2397502

[B36] Mezlini A. M. Smith E. J. Fiume M. Buske O. Savich G. L. Shah S.. (2012). iReckon: simultaneous isoform discovery and abundance estimation from RNA-seq data. Genome Res. 23, 519–529. 10.1101/gr.142232.112 23204306 PMC3589540

[B37] Moran G. P. Coleman D. C. Sullivan D. J. (2010). Comparative genomics and the evolution of pathogenicity in human pathogenic fungi. Eukaryot Cell 10, 34–42. 10.1128/EC.00242-10 21076011 PMC3019795

[B38] Pan Q. Shai O. Lee L. J. Frey B. J. Blencowe B. J. (2008). Deep surveying of alternative splicing complexity in the human transcriptome by high-throughput sequencing. Nat. Genet. 40, 1413–1415. 10.1038/ng.259 18978789

[B39] Parada G. E. Munita R. Cerda C. A. Gysling K. (2014). A comprehensive survey of non-canonical splice sites in the human transcriptome. Nucleic Acids Res. 42, 10564–10578. 10.1093/nar/gku744 25123659 PMC4176328

[B40] Pertea M. Kim D. Pertea G. M. Leek J. T. Salzberg S. L. (2016). Transcript-level expression analysis of RNA-seq experiments with HISAT, StringTie and Ballgown. Nat. Protoc. 11, 1650–1667. 10.1038/nprot.2016.095 27560171 PMC5032908

[B41] Pertea M. Pertea G. M. Antonescu C. M. Chang T. C. Mendell J. T. Salzberg S. L. (2015). StringTie enables improved reconstruction of a transcriptome from RNA-seq reads. Nat. Biotechnol. 33, 290–295. 10.1038/nbt.3122 25690850 PMC4643835

[B42] Pohl M. Bortfeldt R. H. Grützmann K. Schuster S. (2013). Alternative splicing of mutually exclusive exons—a review. Biosystems 114, 31–38. 10.1016/j.biosystems.2013.07.003 23850531

[B43] Prevorovský M. Hálová M. Abrhámová K. Libus J. Folk P. (2016). Workflow for Genome-Wide Determination of Pre-mRNA Splicing Efficiency from Yeast RNA-seq Data. BioMed Res. Int. 2016, 1–9. 10.1155/2016/4783841 28050562 PMC5168555

[B44] Priebe S. Kreisel C. Horn F. Guthke R. Linde J. (2015). FungiFun2: a comprehensive online resource for systematic analysis of gene lists from fungal species. Bioinformatics 31, 445–446. 10.1093/bioinformatics/btu627 25294921 PMC4308660

[B45] Roberts A. Pimentel H. Trapnell C. Pachter L. (2011). Identification of novel transcripts in annotated genomes using RNA-Seq. Bioinformatics 27, 2325–2329. 10.1093/bioinformatics/btr355 21697122

[B46] Robinson J. T. Thorvaldsdóttir H. Winckler W. Guttman M. Lander E. S. Getz G. Mesirov J. P. (2011). Integrative genomics viewer. Nat. Biotechnol. 29, 24–26. 10.1038/nbt.1754 21221095 PMC3346182

[B47] Schreiber K. Csaba G. Haslbeck M. Zimmer R. (2015). Alternative splicing in next generation sequencing data of *Saccharomyces cerevisiae*. PLoS One, 10:e0140487. 10.1371/journal.pone.0140487 26469855 PMC4607428

[B48] Seelbinder B. Wolf T. Guthke R. Linde J. (2018). R Geo2rnaseq :: Anaconda Cloud. Jena. Available online at: https://anaconda.org/xentrics/r-geo2rnaseq

[B49] Shen S. Park J. W. Lu Z. X. Lin L. Henry M. D. Wu Y. N.. (2014). rMATS: robust and flexible detection of differential alternative splicing from replicate RNA-Seq data. Proc. Natl. Acad. Sci. U.S.A. 111, E5593–E5601. 10.1073/pnas.1419161111 25480548 PMC4280593

[B50] Skrzypek M. S. Binkley J. Binkley G. Miyasato S. R. Simison M. Sherlock G. (2017). The *Candida* Genome Database (CGD): incorporation of assembly 22, systematic identifiers and visualization of high throughput sequencing data. Nucleic Acids Res. 45, D592–D596. 10.1093/nar/gkw924 27738138 PMC5210628

[B51] Stajich J. E. Berbee M. L. Blackwell M. Hibbett D. S. James T. Y. Spatafora J. W.. (2009). The fungi. Curr. Biol. 19. 10.1016/j.cub.2009.07.004 19788875 PMC2913116

[B52] Steijger T. Abril J. F. Engström P. G. Kokocinski F. Akerman M. Alioto T.. (2013). Assessment of transcript reconstruction methods for RNA-seq. Nat. Methods 10, 1177–1184. 10.1038/nmeth.2714 24185837 PMC3851240

[B53] Supek F. Bošnjak M. Škunca N. Šmuc T. (2011). Revigo summarizes and visualizes long lists of gene ontology terms. PLoS ONE 6:e21800. 10.1371/journal.pone.0021800 21789182 PMC3138752

[B54] Trapnell C. Hendrickson D. G. Sauvageau M. Goff L. Rinn J. L. Pachter L. (2012a). Differential analysis of gene regulation at transcript resolution with RNA-seq. Nat. Biotechnol. 31, 46—53. 10.1038/nbt.2450 23222703 PMC3869392

[B55] Trapnell C. Roberts A. Goff L. Pertea G. Kim D. Kelley D. R.. (2012b). Differential gene and transcript expression analysis of RNA-seq experiments with TopHat and Cufflinks. Nat. Protoc. 7, 562–578. 22383036 10.1038/nprot.2012.016 PMC3334321

[B56] Urban M. Irvine A. G. Cuzick A. Hammond-Kosack K. E. (2015). Using the pathogen-host interactions database (PHI-base) to investigate plant pathogen genomes and genes implicated in virulence. Front. Plant Sci. 6:605. 10.3389/fpls.2015.00605 26300902 PMC4526803

[B57] Wang E. T. Sandberg R. Luo S. Khrebtukova I. Zhang L. Mayr C.. (2008). Alternative isoform regulation in human tissue transcriptomes. Nature 456, 470–476. 10.1038/nature07509 18978772 PMC2593745

[B58] Wilson D. Thewes S. Zakikhany K. Fradin C. Albrecht A. Almeida R.. (2009). Identifying infection-associated genes of *Candida albicans* in the postgenomic era. FEMS Yeast Res. 9, 688–700. 10.1111/j.1567-1364.2009.00524.x 19473261

