# Supplementary material for: Comparative Study on Alternative Splicing in Human Fungal Pathogens Suggests Its Involvement During Host Invasion
Source: Front Microbiol. 2018 Oct 2;9:2313. doi: 10.3389/fmicb.2018.02313 (PMC6176087; doi:10.3389/fmicb.2018.02313)
Supplement: Supplementary file 1 [file Data_Sheet_1.PDF]

Supplementary Material:
Comparative study on alternative splicing in human fungal pathogens suggests its involvement during host invasion

1 SUPPLEMENTARY DATA

Table S1. Used data sets of studied fungal species. If not stated otherwise, the used data sets are publicly available at NCBI's Gene Expression Omnibus (Edgar, 2002) with the corresponding GSE accession identifier. The conditions are compared as specified in the original publication and briefly explained in brackets.

Species	Data sets response-to-host	Data sets stress
A. fumigatus (A1163)	–	GSE55943 (control 0 min vs oxidative stress 15 min-, 15 min+, 30 min-)<br>GSE55743 (control 0 min vs caspofungin 60 min, 240 min)
C. albicans (SC5314)	GSE56091 (control vs interaction with human cell lines , several time points)<br>GSE38426 (yeast vs induced hyphal morphogenesis)<br>GSE41749 (spore vs induced hyphae)<br>GSE114174 (control 0 min vs blood infection 15 min, 30 min, 60 min, 120 min, 240 min)	GSE49310 (control vs weak organic acids, each for time point ...)<br>GSE73409 (control vs hydroperoxide)
C. glabrata (ATCC2001)	GSE114175 (control 0 min vs blood infection 15 min, 30 min, 60 min, 120 min, 240 min)	GSE74361 (DMSO vs DMSO+KET)<br>GSE61606 (control vs GSNO, pH 4 vs pH 8)
C. parapsilosis (CLIB214)	GSE114177 (control 0 min vs blood infection 15 min, 30 min, 60 min, 120 min, 240 min)	GSE32714 (YPD vs hypoxia, YPD vs BMW)
C. neoformans (KN99α)	–	GSE73203 (control vs induced quorum sensing)<br>GSE32049 (control vs capsule induced conditions)<br>GSE60398 (control vs capsule induced conditions)<br>GSE69532 (control vs capsule induced conditions)
L. corymbifera (JMRC:FSU:9682)	GSE114628 (interaction with murine macrophage after 0 and 16 h)	PRJEB3978 [1] (control vs hypoxia; control vs iron depletion)
H. capsulatum (G217B)	GSE68705 (yeast vs hyphal growth)<br>GSE68706 (yeast vs hyphal growth)	–

[1] This data set is stored at ENA (https://www.ebi.ac.uk/ena/data/view/PRJEB3978)

Table S2. Number of expressed transcripts and genes with a TPM value > 1. The average values of all samples are used. The values of *C. albicans* refer to the haploid gene annotation.

Species	Control		Response-to-host		Stress	
	# genes	# transcripts	# genes	# transcripts	# genes	# transcripts
C. albicans	5624	5942	5673	5972	5683	5975
C. parapsilosis	5743	5784	5127	5144	5718	5751
C. glabrata	4717	4760	4627	4648	4839	4891
A. fumigatus	8384	11320	–	–	8424	11236
H. capsulatum	8984	11570	8317	10439	–	–
C. neoformans	5866	6597	–	–	6415	7219
L. corymbifera	8853	10408	8144	9277	8760	10322

Table S3: Genes with AS based on the prediction of the four AS analysis tools DEXSeq (DX), DiffSplice (DS), MATS (MA) and MISO (MI). In each case, a p-value or FDR of 0.01 is used. The data sets are sorted by species and response-to-host (H) and stress (S) contidions. C indicates the control sample of the corresponding experiment. The filter involves exclusion of genes without introns (w/o intr), too low expression (low exp), and no detectable change in coverage (no ch). The resulting genes are filtered manually (man).

Sp.	Data set	Compared conditions	AS tools DX	DS	MA	MI	all	filter w/o intr	low exp	no ch	man
Af	GSE55743^S	CvsCas60m	628	8	0	0	635	634	616	487	39
		CvsCas240m	70	1	0	0	71	71	70	65	8
	GSE55943^S	0minvs15min-	33	0	3	0	34	34	31	25	10
		0minvs30min-	25	2	0	0	27	27	25	20	5
		0minvs15min+	7	3	0	0	10	10	8	8	1
Ca	GSE38426^H	yeastvshyphae	0	234	0	0	234	133	95	73	4
	GSE41749^H	YPDvsYPS	6	175	0	0	178	86	71	65	11
	GSE56091^H	90mHCvsinf	1	0	0	0	1	1	1	1	0
		5hHCvsinf	0	1	0	0	1	1	1	1	0
		8hHCvsinf	0	0	0	0	0	0	0	0	0
		90mOCvsinf	2	0	0	0	2	2	2	2	0
		5hOCvsinf	1	0	0	0	1	1	0	0	0
		8hOCvsinf	2	0	0	0	2	2	1	1	0
	GSE114174^H	0minvs15min	0	0	0	0	0	0	0	0	0
		0minvs30min	0	0	0	0	0	0	0	0	0
		0minvs60min	1	2	0	0	3	2	1	1	1
		0minvs120min	5	0	0	0	5	5	3	3	0
		0minvs240min	0	0	0	0	0	0	0	0	0
	GSE49310^S	C.T1vsHCl.T1	0	0	0	0	0	0	0	0	0
		C.T2vsHCl.T2	0	0	0	0	0	0	0	0	0
		C.T3vsHCl.T3	0	0	0	0	0	0	0	0	0
		C.T4vsHCl.T4	0	0	0	0	0	0	0	0	0
		C.T1vsA.T1	0	0	0	0	0	0	0	0	0
		C.T2vsA.T2	38	1	0	0	38	38	38	37	3
		C.T3vsA.T3	2	2	0	0	4	3	3	3	0
		C.T4vsA.T4	37	1	0	0	38	37	35	32	2
		C.T1vsB.T1	1	0	0	0	1	1	1	1	1
		C.T2vsB.T2	1	231	0	0	231	153	100	79	5
		C.T3vsB.T3	1	3	0	0	4	3	2	1	1
		C.T4vsB.T4	3	13	0	0	13	10	8	3	1
		C.T1vsL.T1	2	208	0	0	208	130	94	71	5
		C.T2vsL.T2	3	4	0	0	7	6	5	3	0

		C.T3vsL.T3	2	50	0	0	51	39	33	28	1
		C.T4vsL.T4	2	38	0	0	40	26	20	14	1
		C.T1vsP.T1	3	2	0	0	5	5	5	5	3
		C.T2vsP.T2	10	38	0	0	44	37	31	26	3
		C.T3vsP.T3	3	1	0	0	4	4	3	3	2
		C.T4vsP.T4	3	7	0	0	9	5	4	4	3
	GSE73409^S	wtuvswtt	4	425	0	0	425	204	101	90	9
Cg	GSE114175^H	0minvs15min	0	0	0	0	0	0	0	0	0
		0minvs30min	3	1	0	0	4	4	2	1	0
		0minvs60min	3	0	0	0	3	3	2	2	0
		0minvs120min	0	2	0	0	2	2	1	1	0
		0minvs240min	4	25	1	0	29	26	1	1	0
	GSE61606^S	pH4vspH8	16	1	0	0	17	17	14	12	0
		CvsGSNO	34	10	0	0	43	40	22	19	1
	GSE74361^S	DvsDK	34	5	0	0	39	37	19	19	1
Cp	GSE114177^H	0minvs15min	4	0	0	0	4	4	2	2	1
		0minvs30min	2	0	0	0	2	2	1	0	0
		0minvs60min	4	0	0	0	4	4	2	2	0
		0minvs120min	1	0	0	0	1	1	0	0	0
		0minvs240min	0	0	0	0	0	0	0	0	0
	GSE32714^S	YPD21vsBMW21	3	38	0	0	41	36	34	34	10
		YPD21vsYPD01	6	0	0	0	6	6	6	6	2
Cn	GSE32049^S	30Cvs37C	1018	1	0	11	1030	988	27	18	1
	GSE60398^S	30Cvs37C90m	252	3	4	71	311	287	110	66	4
		30Cvs37C180m	369	4	11	71	420	386	175	115	5
		30Cvs37C480m	780	10	6	82	826	781	411	250	9
		30Cvs37C1440m	4189	7	10	152	4205	4060	1636	1172	30
	GSE69532^S	30Cvs37C90m	5525	507	3	249	5656	5395	832	644	31
		30Cvs37C1440m	1579	2665	1	88	3587	3488	37	31	0
	GSE73203^S	WTvssatExpr1	1481	11	0	0	1489	1461	17	14	5
		WTlogvssatExpr2	105	957	0	0	1054	1021	6	1	0
Lc	GSE114628^H	Cvsinfect	24	12	0	0	36	36	8	6	1
	PRJEB3978^S	CvsHY	353	2	12	0	360	360	253	88	21
		CvsSID	23	1	2	0	25	25	19	4	2
Hc	GSE68705^H	yeastvshyphae	146	2	0	0	148	148	81	53	3
	GSE68706^H	yeastvshyphae	2090	486	0	0	2418	2410	2006	1440	233

Table S4. Unique predicted genes undergoing AS per condition compared to the corresponding control.

Species	Uniquely predicted AS genes		Overlap
	Response-to-host	Stress	
C. albicans	16	21	6 [2]
C. parapsilosis	1	12	0
C. glabrata	0	2	0
A. fumigatus	–	59	–
H. capsulatum	236	–	–
C. neoformans	–	75	–
L. corymbifera	1	23	0

Table S5. AS pattern in predicted DETs devided into condition groups and compared to the corresponding control. Last column: AS pattern with accordance to Stringtie results summarized for both conditions.

Sp.	Response-to-host						Stress						Stringtie-predicted
	A3S	A5S	AFE	ALE	ES	IR	A3S	A5S	AFE	ALE	ES	IR	
Ca	2		3			11	4	1	7			13	37 %
Cp						1			2	1		10	8 %
Cg							1					1	50 %
Af							19	12	12	6	4	21	81 %
Hc	45	32	12	2	5	196							48 %
Cn							4		51	11	1	14	61 %
Lc			1				3		3			35	66 %

[2] Common genes: C1_06130C, C3_04590W, C3_06800C, C6_01820C, C7_00110W, CR_06690C

Table S6: DEGs and spliceosomal genes of each data set. The number of significant DEGs is based on DESeq2 with an adjusted p-value < 0.01 and a log fold change > 1. DEGs with a mean TPM value < 1 are considered as not expressed and are excluded. The spliceosomal genes within the set of DEGs and the direction of expression change from control to treatment are counted. The identified DETs are compared to all DEGs and all spliceosomal genes (the last two columns). The data sets are sorted by response-to-host (H) and stress (S) conditions. C indicates the control sample. No spliceosomal genes are annotated for the used strain of *H. capsulatum*.

Sp.	Data set	Compared	DEGs	spliceo-some DEGs	thereof up-regul.	overlap DETs with DEGs	spliceo-some
Af	GSE55743^S	CvsCas60m	434	0	0	2	1
		CvsCas240m	421	0	0	2	0
	GSE55943^S	0minvs15min-	149	0	0	1	0
		0minvs30min-	789	1	1	3	0
		0minvs15min+	44	0	0	0	0
Ca	GSE38426^H	yeastvshyphae	389	8	8	0	0
	GSE41749^H	YPDvsYPS	531	16	16	0	0
	GSE56091^H	90mHCvsinf	0	0	0	0	0
		5hHCvsinf	0	0	0	0	0
		8hHCvsinf	0	0	0	0	0
		90mOCvsinf	0	0	0	0	0
		5hOCvsinf	0	0	0	0	0
		8hOCvsinf	0	0	0	0	0
	GSE114174^H	0minvs15min	398	9	9	0	0
		0minvs30min	812	17	17	0	0
		0minvs60min	740	18	18	0	0
		0minvs120min	400	7	7	0	0
		0minvs240min	337	3	3	0	0
	GSE49310^S	C.T1vsHCl.T1	11	1	1	0	0
		C.T2vsHCl.T2	11	1	1	0	0
		C.T3vsHCl.T3	6	2	2	0	0
		C.T4vsHCl.T4	10	1	1	0	0
		C.T1vsA.T1	171	3	3	0	0
		C.T2vsA.T2	562	21	21	0	0
		C.T3vsA.T3	236	7	7	0	0
		C.T4vsA.T4	436	15	15	0	0
		C.T1vsB.T1	289	9	9	0	0
		C.T2vsB.T2	527	12	12	0	0
		C.T3vsB.T3	507	12	12	0	0
		C.T4vsB.T4	498	13	13	0	0

		C.T1vsL.T1	193	4	4	0	0
		C.T2vsL.T2	335	12	12	0	0
		C.T3vsL.T3	250	5	5	0	0
		C.T4vsL.T4	182	6	6	0	0
		C.T1vsP.T1	351	10	10	0	0
		C.T2vsP.T2	608	14	14	0	0
		C.T3vsP.T3	481	12	12	0	0
		C.T4vsP.T4	424	11	11	0	2
	GSE73409^S	wtuvswtt	871	28	28	0	0
Cg	GSE114175^H	0minvs15min	33	0	0	0	0
		0minvs30min	40	0	0	0	0
		0minvs60min	106	1	1	0	0
		0minvs120min	79	0	0	0	0
		0minvs240min	56	0	0	0	0
	GSE61606^S	pH4vspH8	383	3	3	0	0
		CvsGSNO	1105	18	18	0	0
	GSE74361^S	DvsDK	1301	33	33	0	0
Cp	GSE114177^H	0minvs15min	968	4	4	0	0
		0minvs30min	695	3	3	0	0
		0minvs60min	343	0	0	0	0
		0minvs120min	71	0	0	0	0
		0minvs240min	76	0	0	0	0
	GSE32714^S	YPD21vsBMW21	19	0	0	0	0
		YPD21vsYPD01	333	0	0	0	0
Cn	GSE32049^S	30Cvs37C	61	0	0	0	0
	GSE60398^S	30C0mvs37C90m	547	0	0	0	0
		30C0mvs37C180m	626	1	1	0	0
		30C0mvs37C480m	777	0	0	0	0
		30C0mvs37C1440m	1074	0	0	3	1
	GSE69532^S	30C0mvs37C90m	1302	3	3	4	0
		30C0mvs37C1440m	1422	3	3	0	0
	GSE73203^S	WTvssatExpr1	2008	0	0	0	0
		WTlogvssatExpr2	33	24	24	0	0
Lc	GSE114628^H	Cvsinfect	277	0	0	0	0
	PRJEB3978^S	CvsHY	627	1	1	5	0
		CvsSID	22	0	0	0	0
Hc	GSE68705^H	yeastvshyphae	922	(0	0)	1	(0)
	GSE68706^H	yeastvshyphae	1195	(0	0)	51	(0)

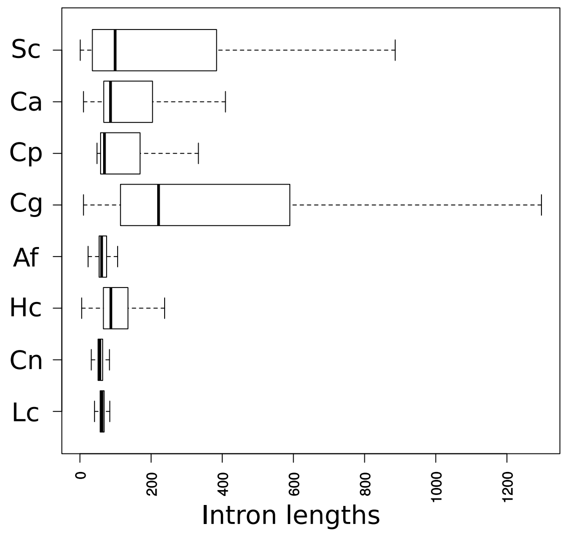

Figure S1. Boxplot of lengths distribution of introns of the studied fungal species including baker's yeast *S. cerevisiae* for comparisons.

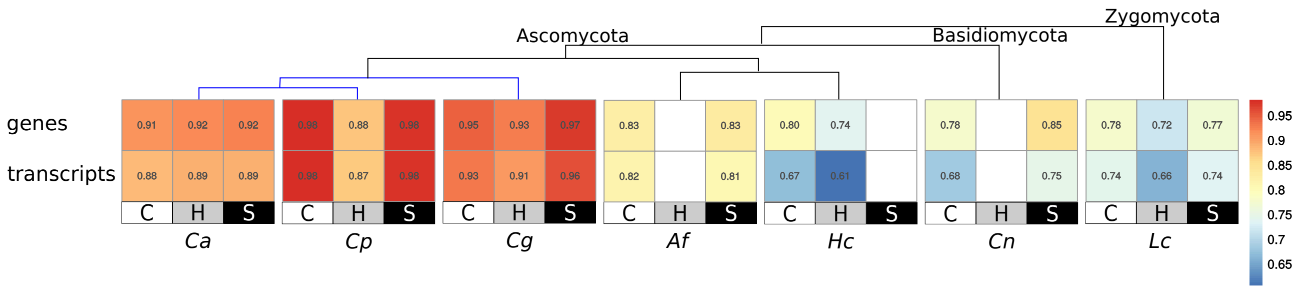

Figure S2. Number of expressed genes and isoforms relative to the total number of genes and transcripts per species. The results are divided into the condition groups control (C), response-to-host (H) and stress (S).

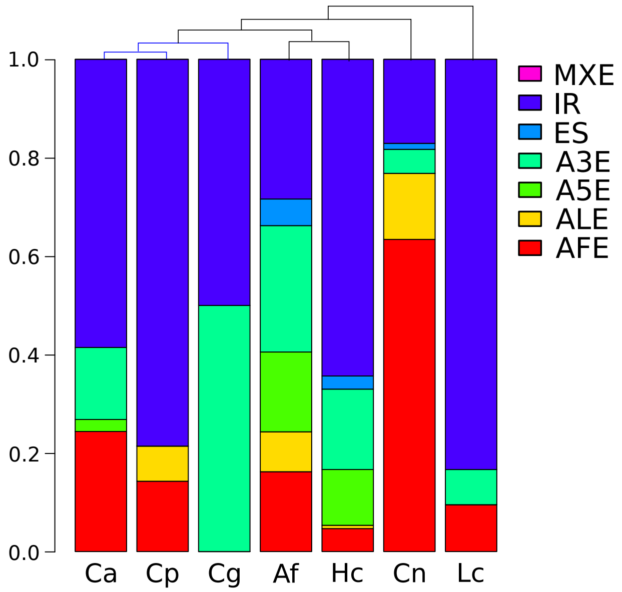

Figure S3. Identified AS patterns of detected DETs per species merged for response-to-host and stress conditions.

REFERENCES

Edgar, R. (2002). Gene expression omnibus: Ncbi gene expression and hybridization array data repository. *Nucleic Acids Res* 30, 207–210

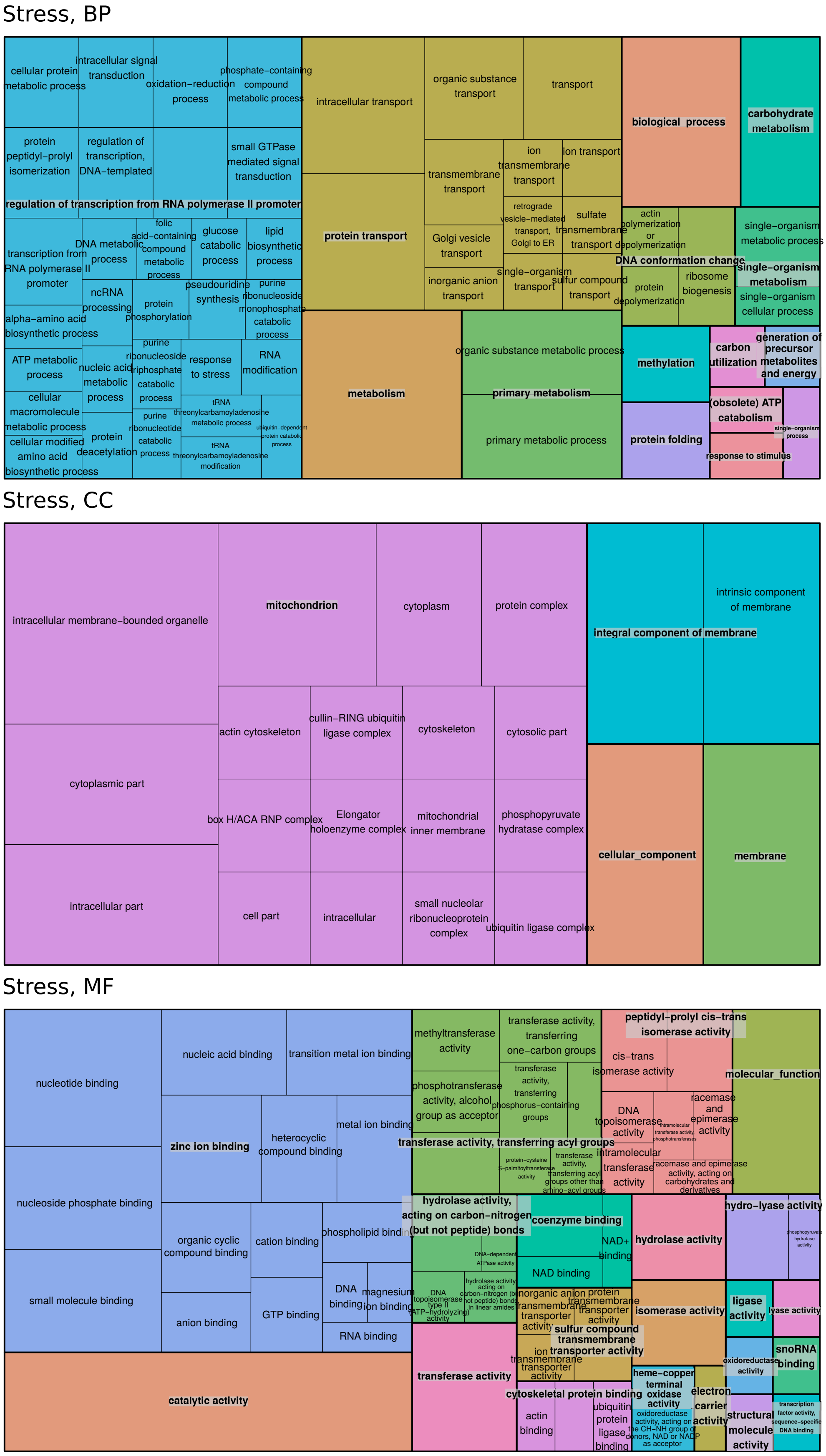

Figure S4. Tree maps of GO terms corresponding to DETS of *A. fumigatus* for stress conditions, for the GO term categories biological process (BP), cellular component (CC), molecular function (MF).

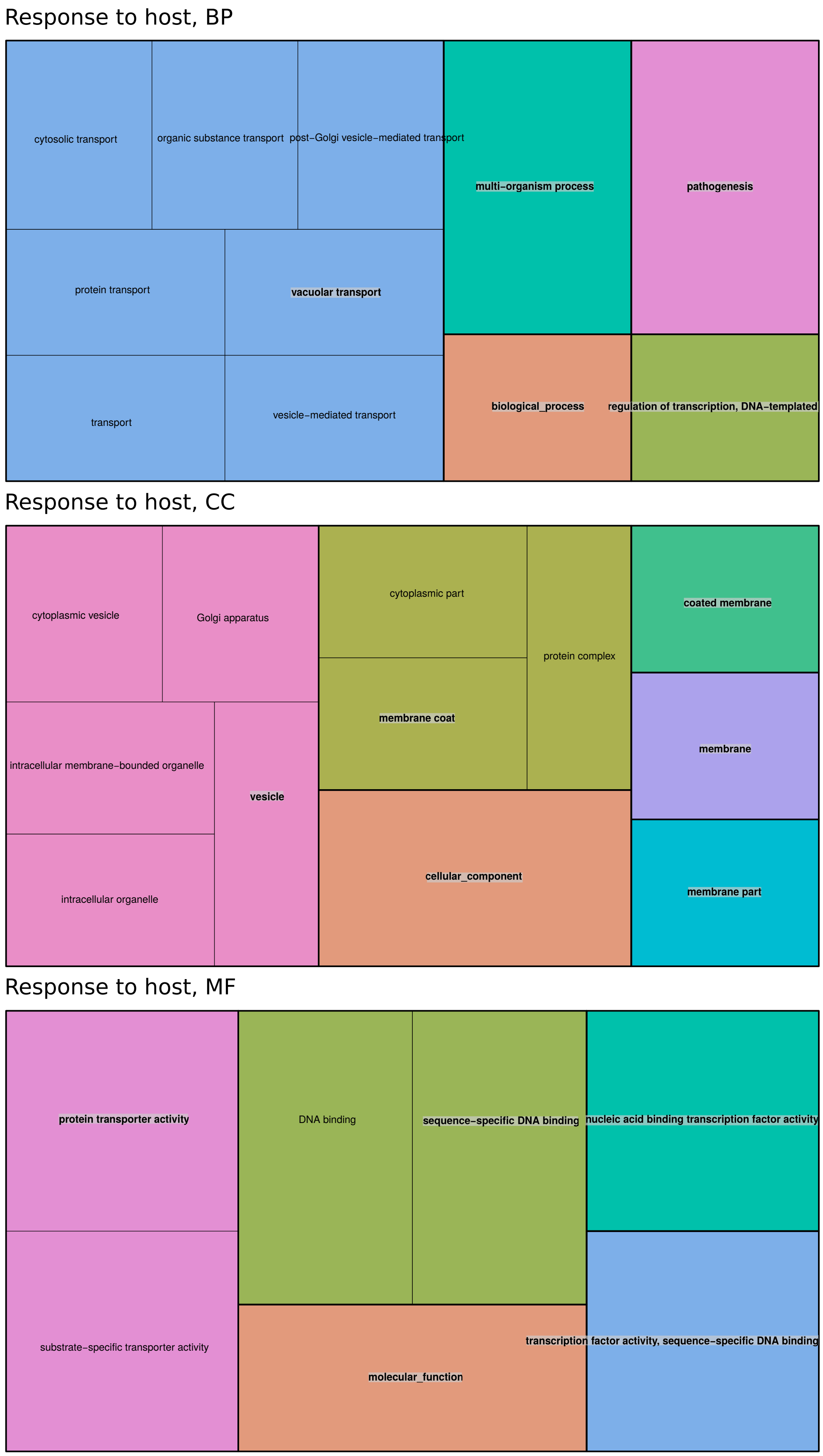

Figure S5. Tree maps of GO terms corresponding to DETS of *C. albicans* for response-to-host conditions, for the GO term categories biological process (BP), cellular component (CC), molecular function (MF).

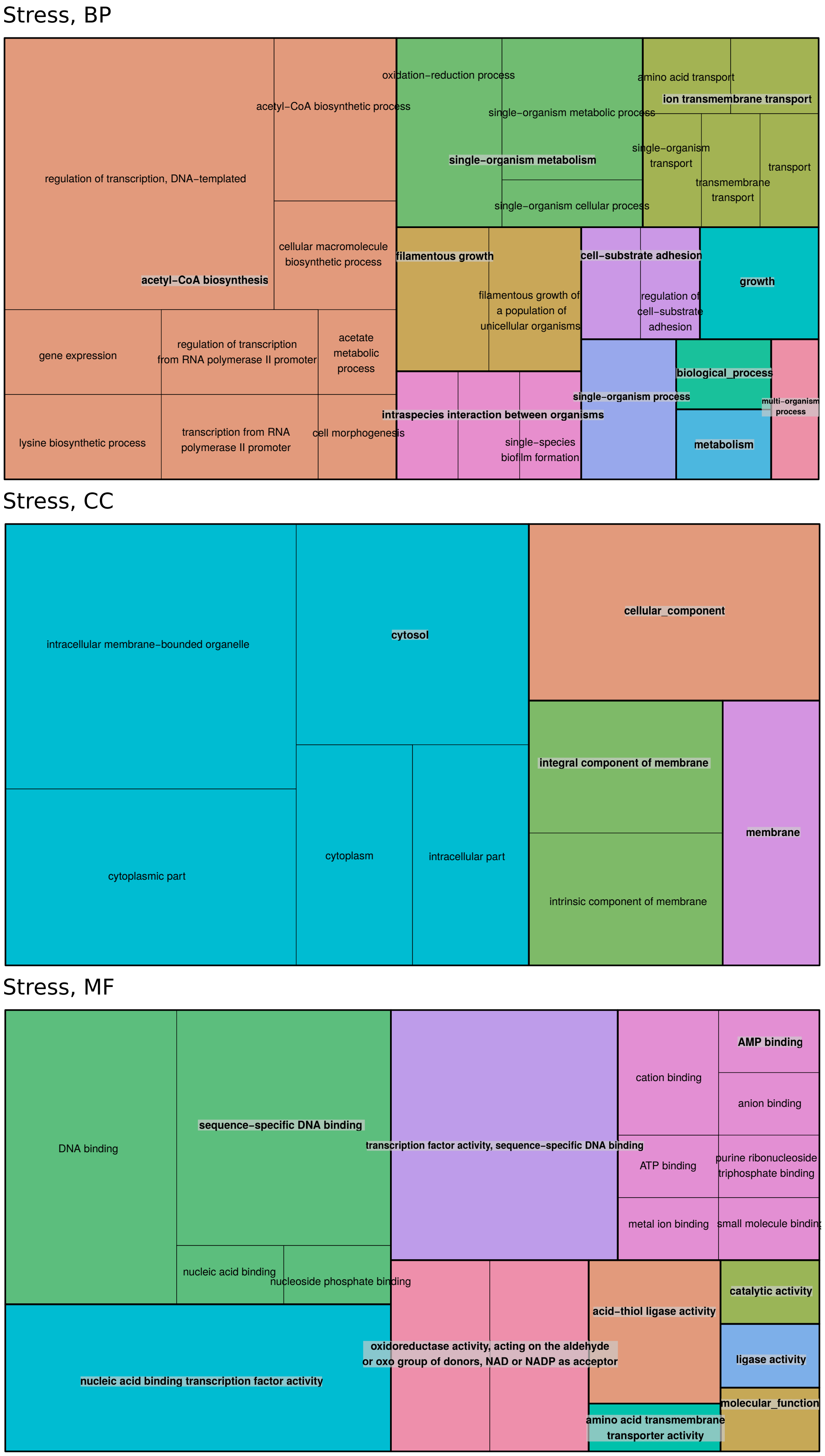

Figure S6. Tree maps of GO terms corresponding to DETS of *C. albicans* for stress conditions, for the GO term categories biological process (BP), cellular component (CC), molecular function (MF).

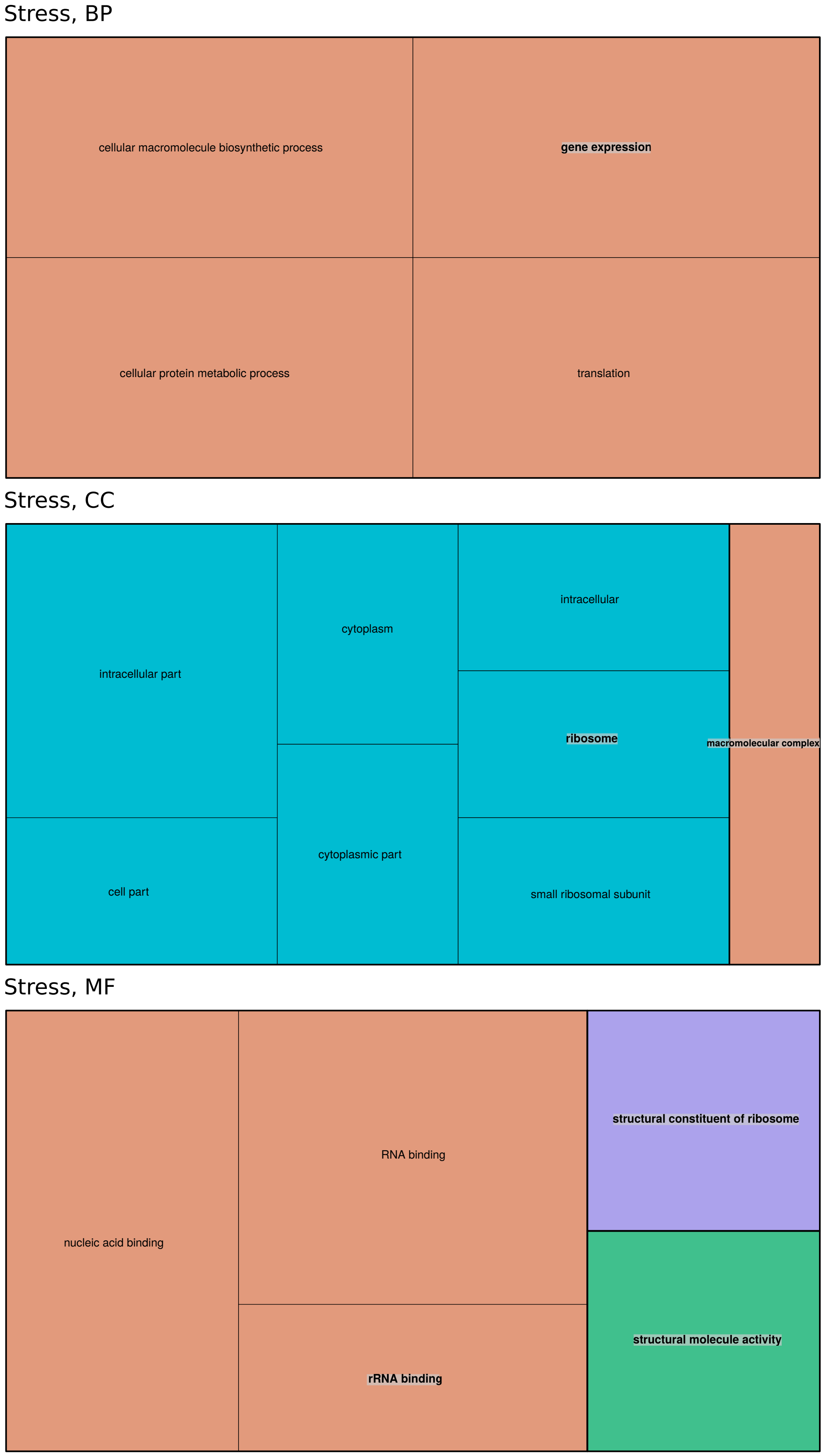

Figure S7. Tree maps of GO terms corresponding to DETS of *C. glabrata* for stress conditions, for the GO term categories biological process (BP), cellular component (CC), molecular function (MF).

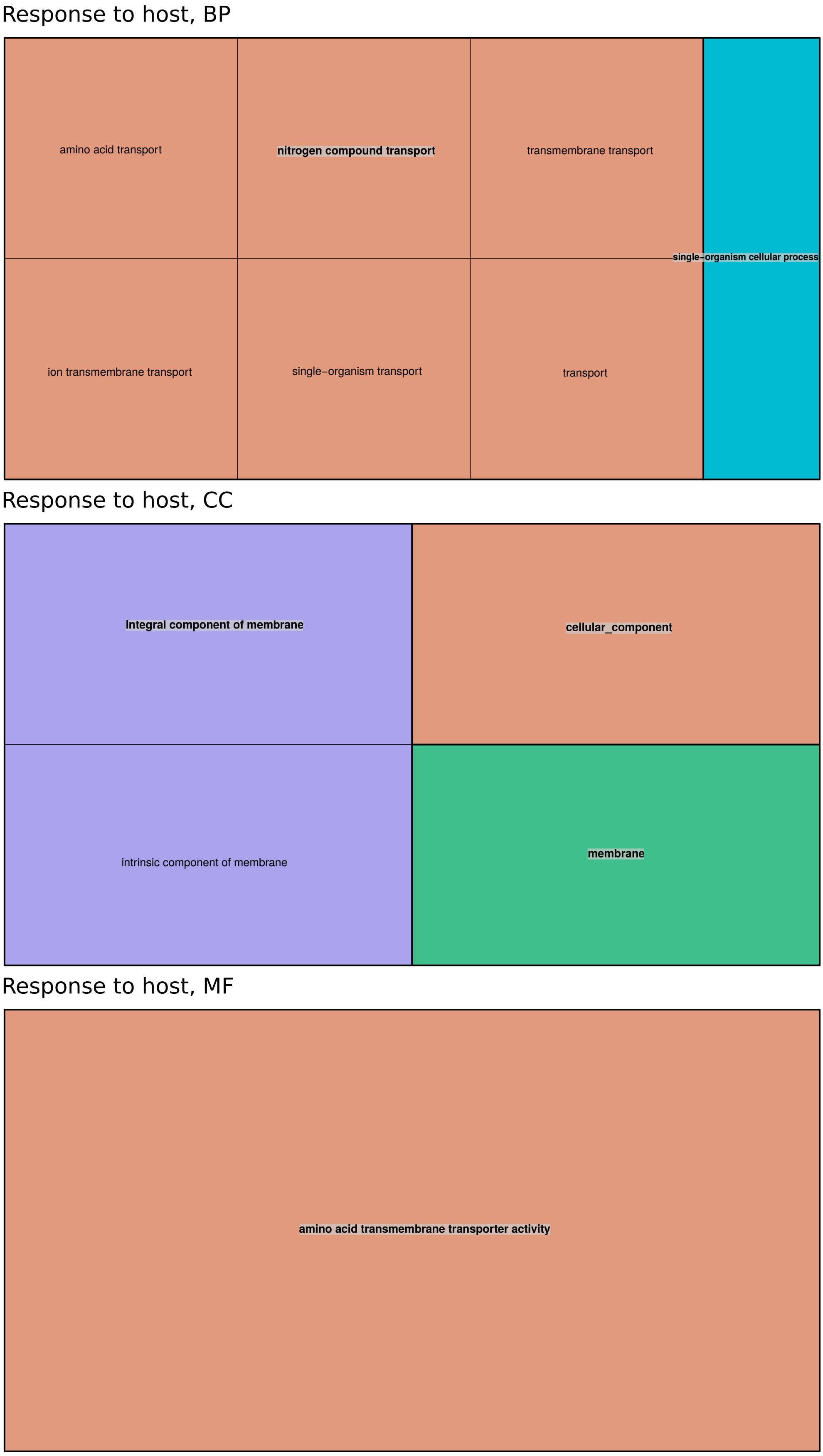

Figure S8. Tree maps of GO terms corresponding to DETS of *C. parapsilosis* for response-to-host conditions, for the GO term categories biological process (BP), cellular component (CC), molecular function (MF).

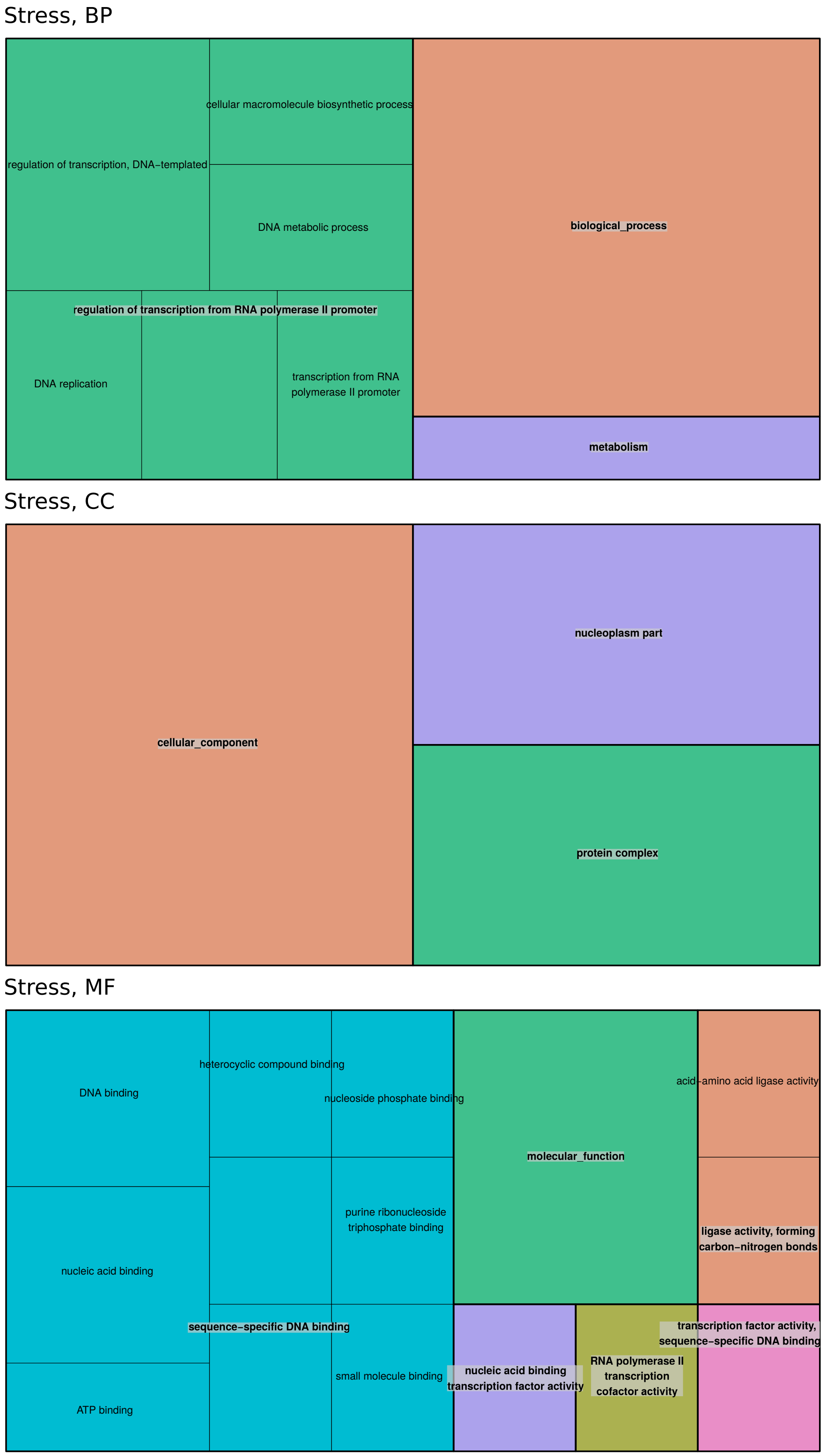

Figure S9. Tree maps of GO terms corresponding to DETS of *C. parapsilosis* for stress conditions, for the GO term categories biological process (BP), cellular component (CC), molecular function (MF).

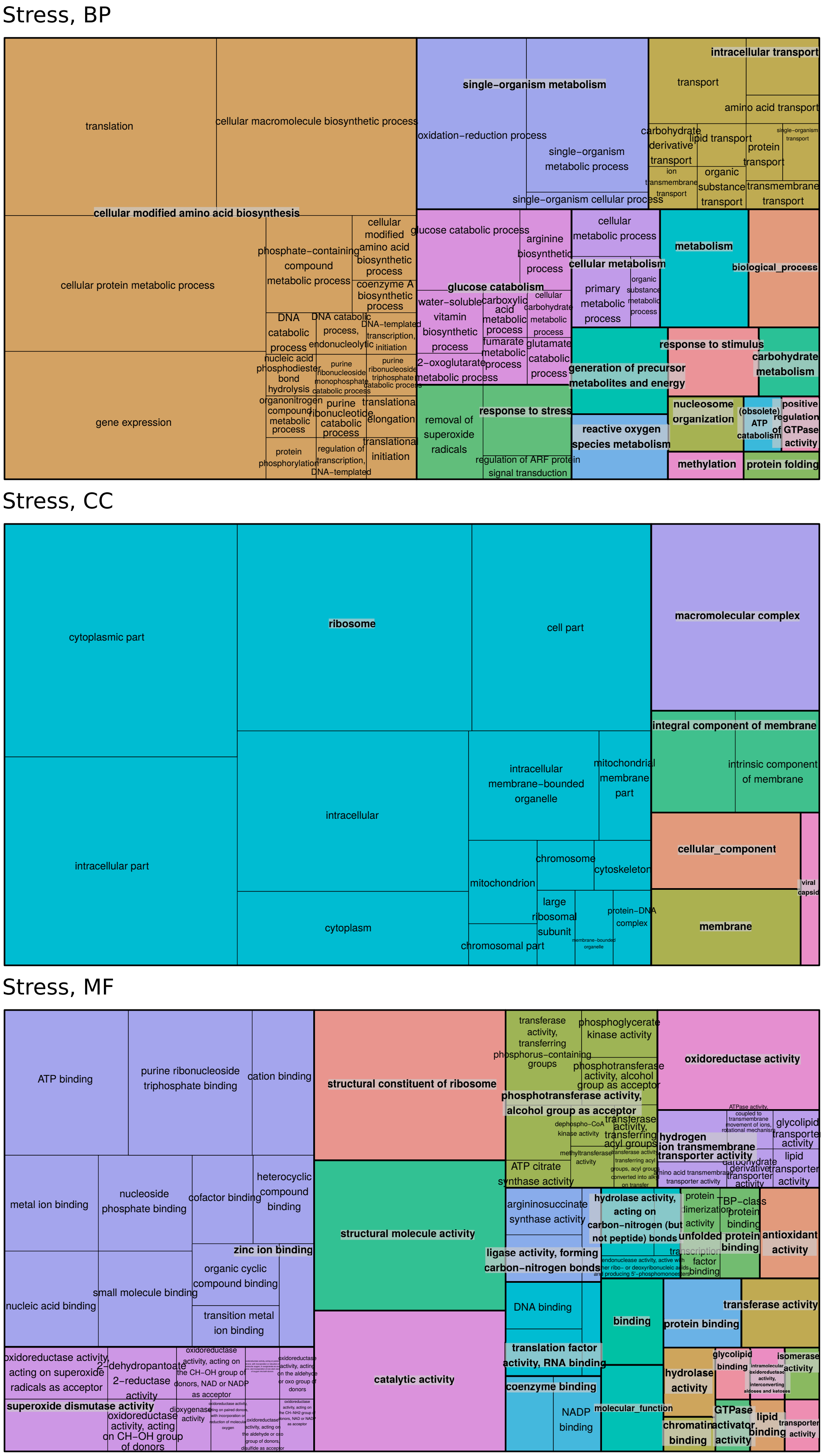

Figure S10. Tree maps of GO terms corresponding to DETS of *C. neoformans* for stress conditions, for the GO term categories biological process (BP), cellular component (CC), molecular function (MF).

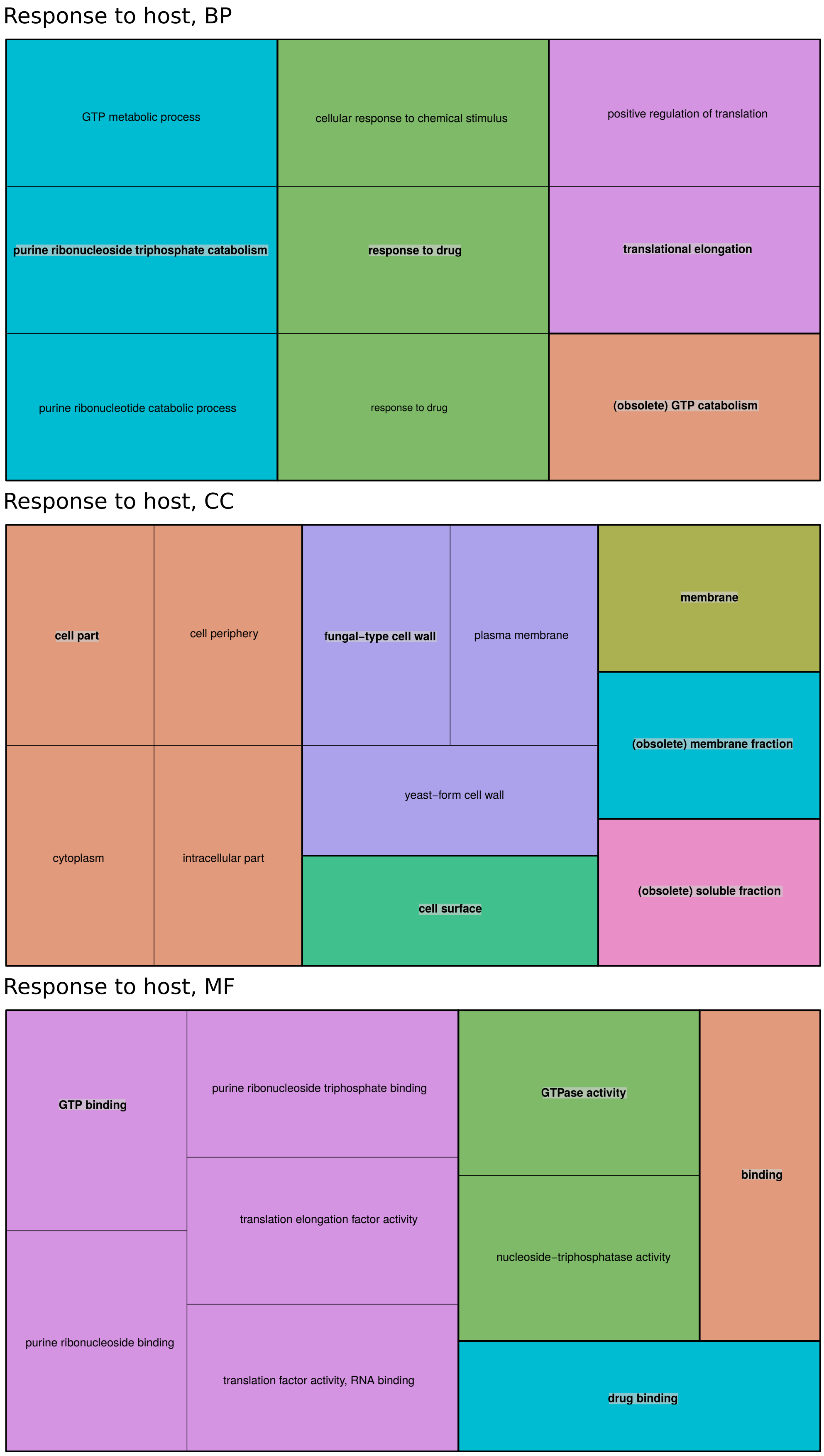

Figure S11. Tree maps of GO terms corresponding to DETS of *L. corymbifera* for response-to-host conditions, for the GO term categories biological process (BP), cellular component (CC), molecular function (MF).

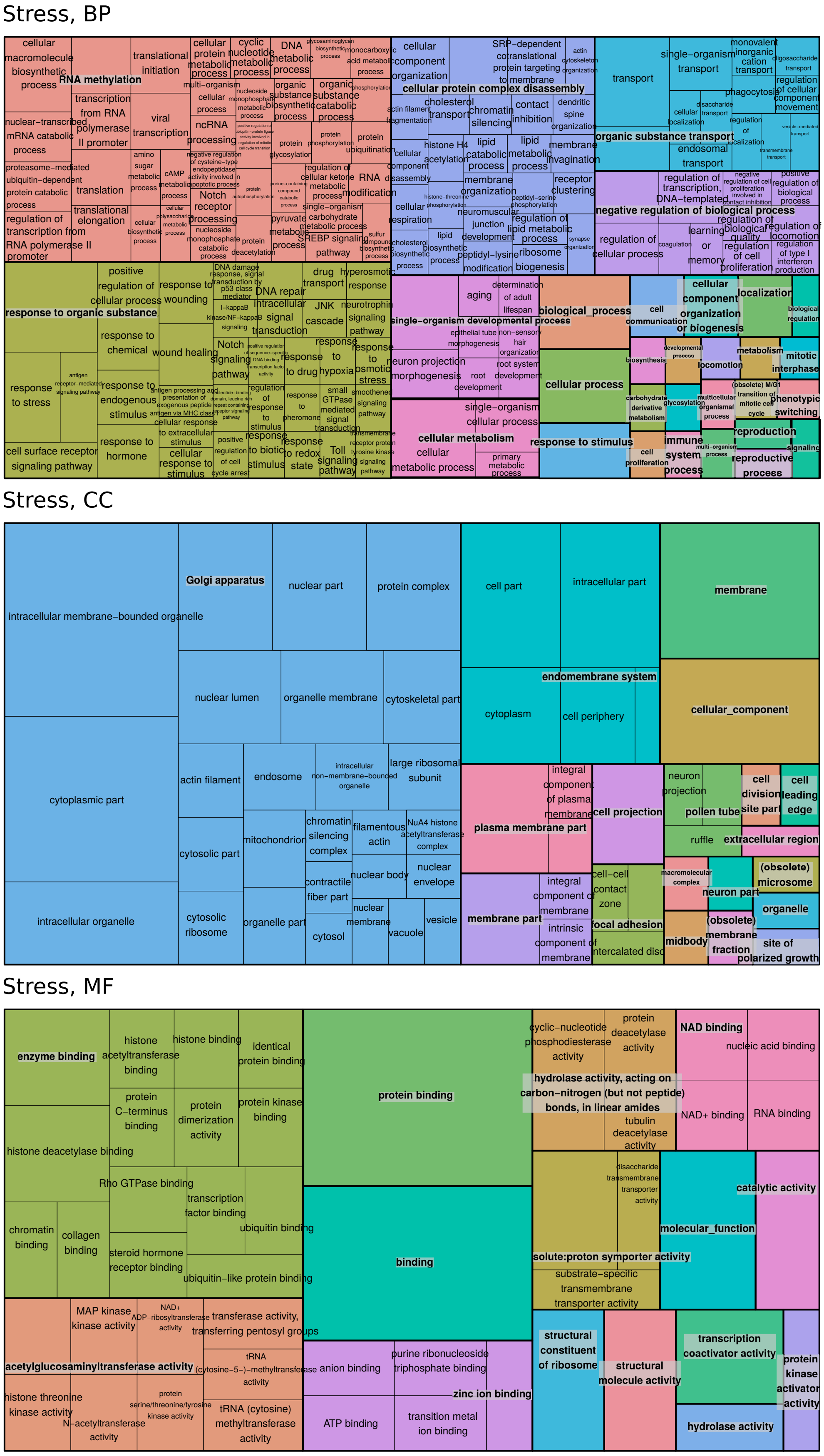

Figure S12. Tree maps of GO terms corresponding to DETS of *L. corymbifera* for stress conditions, for the GO term categories biological process (BP), cellular component (CC), molecular function (MF).
